# Algorithmic Approaches for Assessing Multiscale Irreversibility in Time Series: Review and Comparison

**DOI:** 10.3390/e27020126

**Published:** 2025-01-25

**Authors:** Massimiliano Zanin, David Papo

**Affiliations:** 1 Instituto de Física Interdisciplinar y Sistemas Complejos IFISC (CSIC-UIB), Campus UIB, 07122 Palma de Mallorca, Spain; 2 Department of Neuroscience and Rehabilitation, Section of Physiology, University of Ferrara, 44121 Ferrara, Italy; 3 Center for Translational Neurophysiology, Fondazione Istituto Italiano di Tecnologia, 44121 Ferrara, Italy

**Keywords:** irreversibility, time-reversal symmetry breaking, multiscale

## Abstract

Many physical and biological phenomena are characterized by time asymmetry, and are referred to as irreversible. Time-reversal symmetry breaking is in fact the hallmark of systems operating away from equilibrium and reflects the power dissipated by driving the system away from it. Time asymmetry may manifest in a wide range of time scales; quantifying irreversibility in such systems thus requires methods capable of detecting time asymmetry in a multiscale fashion. In this contribution we review the main algorithmic solutions that have been proposed to detect time irreversibility, and evaluate their performance and limitations when used in a multiscale context using several well-known synthetic dynamical systems. While a few of them have a general applicability, most tests yield conflicting results on the same data, stressing that a “one size fits all” solution is still to be achieved. We conclude presenting some guidelines for the interested practitioner, as well as general considerations on the meaning of multiscale time irreversibility.

## 1. Introduction

Time-reversal symmetry is the symmetry under the time flow transformation t↦−t of a physical law or of its realizations. Processes which are invariant under this transformation look the same as the flow of time is reversed and are called reversible, while those which are not are said to be irreversible. Reversible processes and their time-reversed version are indistinguishable, at least in a statistical sense. Conversely, the larger the difference between a process and its time-reversed version the easier it is to distinguish them. Thus, time-reversal symmetry quantifies the extent to which it is possible to distinguish a preferred time direction of a time-ordered realisation of a stationary stochastic process [1]. Irreversibility is a characteristic feature of systems, e.g., living or more generally active matter, operating away from equilibrium [2]. These systems use free energy to perform work or to store energy. For instance, in biological systems mechanochemical reactions at microscopic scales use free energy through metabolism to drive large-scale rearrangements and functions [3]. Thus, thermodynamic irreversibility is a key property of non-equilibrium systems, crucial for maintaining the complex spatio-temporal structure and functions that they support. According to the second law of thermodynamics this transformation is associated with an irreversible increase in entropy of the environment, usually associated with dissipated heat. The thermodynamic rate at which entropy is produced can be understood in terms of asymmetry of temporal disorder, whereby the probability distribution of the forward path decays slower than the corresponding time-reversed path [4,5]. For non-equilibrium steady state systems, the entropy production is proportional to the difference between the Kolmogorov–Sinai entropy of the forward and backward paths, reflecting the fact that forward paths are more ordered than their time-reversed correspondent [4,6]. Thus, measuring irreversibility is in some sense tantamount to quantifying the extent to which a system operates far from equilibrium. Moreover, the higher the entropy lost to dissipation, the more conspicuous the time asymmetry, suggesting that time-reversal symmetry can be used not only as an indicator of whether a system is at equilibrium or not, but also as a quantifier of its distance from such a condition [7]. It is worth noting that while the same basic definition of time-reversal symmetry applies to purely time series and to physical contexts, profound context-dependent differences may arise in the way this may be ascertained. In the former approach, time reversal results from the time-reversal operator itself, and what is evaluated is typically a local function; in the latter, time reversal results from an experimental protocol inversion, and this implies some practicalities, related to the fact that what is evaluated is a probability distribution.

There are various ways in which one can, in principle, determine whether a system lies far from equilibrium and quantify the distance from such a condition [2]. For instance, at thermal equilibrium, the average power spectrum of fluctuations in the unperturbed system is related to the linear response function through the fluctuation–dissipation theorem [8], a condition typically violated in a system operating away from equilibrium, such as neural populations [9,10,11,12,13]. However, this approach requires perturbing the system, which may often not be possible. Moreover, equilibrium fluctuations obey detailed balance of the probability fluxes, meaning that the net current between any pair of states vanishes at long enough times [14,15,16]. Broken detailed balance entails asymmetric transition rates between pairs of microstates, breaking time-reversal symmetry and allowing cycles in the phase space.

From a time series viewpoint, it is important to characterize the properties underlying stochastic processes associated with the time-reversal symmetry and its breakdown [17,18,19] (see [20] for a review). It has been shown that reversibility implies stationarity [18] and that linear Gaussian random processes and static non-linear transformations of such processes are reversible, whereas time irreversibility implies non-linear dynamics, linear or non-linear non-Gaussian processes as possible generative processes [17,18,19]. Indeed, memory breaks time-reversal symmetry, acting as a dissipative force, whereas noise results in a loss of irreversibility [21]. Thus, estimating the degree of irreversibility of a time series implicitly quantifies the degree of non-linear dependences.

One important question has to do with the temporal scale or characteristic time at which time-reversal symmetry emerges [22]. In general, characteristic times provide an order of magnitude of the speed of a process. This is often thought of as a measure of how fast that process approaches equilibrium after some perturbation. To illustrate how a process can be endowed with time scales one may consider a dissipative dynamic process describing the motion of a diffusing macroscopic particle in a configuration space subject to a viscous friction, changing with a time scale $\tau_{m}$, and to an additive random force η(t) drive having a characteristic time $\tau_{\eta }$. If the noise has fast vanishing Gaussian delta-correlated fluctuations, and $\tau_{\eta }\ll \tau_{m}$, the temporal autocorrelation of macroscopic velocity fluctuations C(τ) decays as $exp(-t⁄\tau_{m})$. $\tau_{m}$ is unique and is called characteristic time. The characteristic time $\tau_{m}$, the corresponding correlation time, i.e., the integral of the autocorrelation function over its support, and the correlation length, i.e., the value ξ such that C(t=ξ)=0, endow a given process with a temporal scale and can be understood as measures of how fast the system loses memory of its own past [9].

Various systems are inherently multiscale, i.e., important features appear at multiple scales of time and/or space. Multiscaleness can arise in different contexts and for different reasons. For complex systems, such as macroscopic phenomena, it may be the result of the interplay of multiple processes evolving at different time scales. For instance, biological systems can in general be thought of as driven non-equilibrium systems with chemical reaction networks, each having a characteristic time scale given by its kinetic reaction constant. In other cases, multiscaleness may reflect qualitatively different behaviours at different time scales. A typical example is viscoelasticity, which shows elastic properties at short time scales, and viscosity at long ones. Multiscaleness may also be understood in terms of the relationship between the fluctuations of an unperturbed system and the relaxation properties of that system following a perturbation. For a system at equilibrium, the autocorrelation function in the unperturbed system and the response to an external field conjugate to some observable are related through temperature. In an equilibrium system, any thermometer coupled to a part of the system reads the same temperature [8]. In out-of-equilibrium systems, thermalization happens at widely different time scales simultaneously, within the same region of space [23]. Remarkably, a system may be at equilibrium on one scale and out of equilibrium on another [23]. In such systems, irreversibility may present non-trivial scale-dependence [24,25]. For instance, irreversibility may be undetectable at microscopic scales and appear at mesoscopic ones [14] and vanish again at macroscopic ones [24]. Indeed, complex biological systems such as the brain [26,27] or the heart [28] exhibit scale-dependent time-reversal symmetry breaking. Thus, in a multiscale system, irreversibility defines a relationship between the system’s dissipation time scales and the system’s nonequilibrium activity. As in the single scale case, detailed information about the time scales of nonequilibrium fluctuations could in principle be revealed in various ways, e.g., through departures from the fluctuation–dissipation theorem; however, this usually requires prior information about the mechanisms. In practice, methods designed to detect and quantify time-reversal symmetry breaking resort to coarse-graining procedures that are independent of the intrinsic structure of the system, so that ultimately the system is understood to be irreversible if time-reversal symmetry is broken not only in the original time series but also in coarse-grained versions of the same [29].

While not as widespread as the analysis of the single-scale version, various ways to quantify multiscale irreversibility have been applied in fields ranging from biological systems, including heart rates and dynamics [30,31,32,33,34] and cell metabolism [25], to financial markets [35,36], gas and fluid dynamics [37,38], and technological systems [39].

It is important to note that, from a time series analysis viewpoint, time scales typically take two meanings. On the one hand, when exploring the space of possible time scales ranging from the smallest time unit (sampling rate in an experimental setting) to the observation time, a time scale simply corresponds to a convenient fraction of the latter measured in units of the former. The process through which the space is explored is named coarse-graining. Down-sampling is a particular coarse-graining procedure. On the other hand, an intrinsic time scale of the studied process is the part of the scale space at which a given property features. In the specific case of interest in this paper, it is the particular scale at which the system shows time-reversal symmetry. Note that, while coarse-graining degrades the estimate of a property featuring at a given scale (this is for instance the case of entropy production estimates, which are quantitatively less accurate though, importantly, not qualitatively distorted), it also allows for highlighting multiple scales at which a process can feature a given property.

In the remainder, we test 15 existing algorithmic procedures for detecting irreversibility in time series and quantify their performance in a multiscale context, using several synthetic dynamical systems of well-understood properties and their coarse-grained versions. This work is thus complementary to and expands on Ref. [20], where the same tests were evaluated in a single-scale context. We start by introducing the basic methods, including the irreversibility tests (Section 2.1) and the algorithms for coarse-graining (or downsampling) the time series (Section 2.2). We then present in Section 3 a synthetic model able to generate time series of tuneable irreversibility at specific time scales, thus allowing us to perform a first comparison of the considered tests. Next, we move to some well-known dynamical systems, including the Lorenz dynamical system (Section 4), the asymmetric Weierstrass function (Section 5), two Brownian motion models (Section 6), and two unidimensional discrete chaotic maps (Section 7). This is complemented in Section 8 by an analysis of the multiscale irreversibility of a real-world system, specifically the evolution of Bitcoin prices. In each case we present a review of the evolution of the irreversibility as a function of the considered time scale τ, in addition to other analyses when relevant; full results are also included in Supplementary Material. Finally, in Section 9 we discuss the main results and draw some general conclusions.

## 2. Methods

### 2.1. Irreversibility Tests

For the sake of completeness, in what follows we briefly describe the 15 irreversibility tests considered in this work, presented in alphabetical order. The interested reader can find further information in the provided references, as well as in a previous review on the topic [20]. A synthesis of the tests, with basic references and default parameters, is additionally reported in Table 1. The software implementation of all tests corresponds to the one available at (Irreversibility Tests Library at https://gitlab.com/MZanin/irreversibilitytestslibrary (accessed on 20 December 2024)).

- *BDS*. Brock, Dechert and Scheinkman [40,41,42] proposed a test for low-dimensional chaos based on the calculation of the statistics:
  (1) $$w_{d}\left(r,T\right)=\sqrt{T}\frac{C_{m}\left(r,T\right)-C_{1}{\left(r,T\right)}^{m}}{\sigma_{m}\left(r,T\right)},$$
  with x(t) being a time series of *T* observations, $C_{m}\left(r,T\right)$ is the sample correlation integral at embedding dimension *m* and scaling parameter *r*, and $\sigma_{m}\left(r,T\right)$ is the estimated standard deviation of the statistic under the null hypothesis of independent data. Under such hypothesis, $w_{d}$ is distributed asymptotically as N(0,1). Note that this null hypothesis implies both the absence of low-dimensional chaos, and, relevant for this work, of time asymmetrical dynamics.
- *COP*. Continuous Ordinal Patterns [43] (COPs in short) are an evolution of permutation patterns (which will be described below) that allow the seamless integration of the amplitude of the time series into the analysis, hence addressing one of the main limitations of the latter ones [44,45]. Given a COP and a sub-window of the time series, a distance ϕ between both is calculated. When this process is repeated over the whole time series, the distributions of ϕ for the original and time-reversed time series are expected to be the same in the case of time reversible processes; hence, irreversibility can be tested, e.g., through Kolmogorov–Smirnov two-samples test on the two distributions.
- *Costa Index*. This test was originally proposed as a description of heartbeat dynamics, but later found general applicability [46]. It is based on the comparison of the number of instances in which the time series increases or decreases, i.e., x(t)>x(t+1) vs. x(t)<x(t+1), which are expected to be similar in a time series that is time-reversible. A *p*-value is obtained by comparing the difference in the number of such instances with that expected in surrogate versions of the original time series.
- *DFK*. This test, introduced by Daw, Finney, and Kennel (hence the acronym) [47], is based on partitioning the time series in *n* equiprobable regions, with each one represented by a symbol, for then mapping each time series’ value into one of them. Next, groups of *L* consecutive symbols are merged together to create “words”. Irreversibility is finally assessed by comparing the probability of appearance of each word in the original and time-reversed time series, normally using a $\chi^{2}$ test.
- *Diks*. The Diks’ irreversibility test is based on evaluating whether two sets of vectors, extracted from the original and time-reversed time series, correspond to the same multi-dimensional probability distribution [48]. Specifically, vectors representing subsets of the original time series are extracted, usually non-overlapping sub-windows of embedding dimension *m* and with an embedding delay τ; afterwards, the distance between these and their time-reversed counterpart is evaluated. As a final step, the resulting distances are compared with an unbiased estimator under the null hypothesis of independence.
- *Local CC*. Along with the Visibility Graph method (see below), this test is based on representing time series as complex networks [49], i.e., graphs composed of nodes that correspond to individual data points, pairwise connected when the underlying values fulfil some geometrical rules [50]. In the simplest case, links can be created whenever the line connecting the values corresponding to two nodes is not obstructed by another intermediate point; in other words, when values “can see each other”. The result is called directed Horizontal Visibility Graph (dHVG) [50]. Once such a network is created, it can be analysed in several ways. As proposed in Ref. [51], a possibility is to compare the retarded and advanced local clustering coefficients, i.e., the propensity of the network to form triangles, respectively, backward and forward in time. These two sets of values are then transformed into a *p*-value using a Kolmogorov–Smirnov two-samples test.
- *MS Trends*. This test is based on micro-scale trends, i.e., the slopes of linear fits (or the highest-degree coefficients in polynomial fits) obtained for small overlapping sub-windows of the original time series [52]. As reversing the arrow of time of a series results in a change in the sign in the slope, i.e., from α to −α, a sufficient requirement for time irreversibility is to observe a probability distribution of the slopes not symmetrical with respect to zero. This can easily be tested through a Kolmogorov–Smirnov test. The parameters of the analysis are the length δ of the sub-windows, and the degree *d* of the polynomial fit.
- *Permutation patterns*. Family of tests based on the symbolisation of a time series using permutation patterns, i.e., the rank sequences corresponding to short sub-windows of size *D* of the original series [53,54,55]. The resulting symbol frequencies are then analysed to detect time asymmetries, or deviations with respect to what is observed in the surrogate time series. Many similar tests have been proposed, mostly varying in the way the statistical significance is calculated [56,57,58,59,60]. We here specifically consider what is proposed in Ref. [56], involving the calculation of the difference in the frequencies of each pattern and of its time-reversed version, and the evaluation of the statistical significance of such a difference through a binomial test.
- *Pomeau*. One of the first tests proposed to detect irreversibility in a time series (see also [17,61]) was introduced by Yves Pomeau in 1982 [1]. It is based on calculating a time-asymmetric function on the data, defined as follows:
  (2) $$\psi \left(\tau \right)=\bar{x(t)[x(t+2\tau )-x(t+\tau )](x+3\tau )},$$
  with τ being a lag constant here, set to 1. The obtained value of ψ is then compared to what was obtained in a large set of surrogate time series, in order to obtain an approximated *p*-value.
- *Ramsey*. James B. Ramsey and Philip Rothman proposed a test for irreversibility based on the comparison of the method-of-moments estimators of two sample bicovariances [62], respectively, given by the following:
  $$\begin{array}{c} {\hat{B}}_{2,1}\left(k\right)={\left(T-k\right)}^{-1}\sum_{t=k+1}^{T}x_{t}^{2}x_{t-k}\\ {\hat{B}}_{1,2}\left(k\right)={\left(T-k\right)}^{-1}\sum_{t=k+1}^{T}x_{t}x_{t-k}^{2} \end{array}$$
  Here, *T* is the total length of the time series, and *k* is a parameter defining the lag— i.e., not dissimilar from the τ in the Pomeau’s test. When the time series under analysis is time-reversible, the difference between ${\hat{B}}_{2,1}\left(k\right)$ and ${\hat{B}}_{1,2}\left(k\right)$ tends to zero; hence irreversibility can be evaluated using a *t*-test.
- *Skewness*. This test, initially proposed by Demetris Koutsoyiannis in Ref. [63] and subsequently extended in Ref. [64], is based on considering the original time series x(t) and its differenced version $\tilde{x}\left(t\right)=x_{t}-x_{t-1}$. The skewness (i.e., the degree of asymmetry in the distribution) of these two time series is calculated, respectively denoted as $C_{s}$ and ${\tilde{C}}_{s}$; finally, a time-irreversibility index is defined as $a={\tilde{C}}_{s}/C_{s}$. As discussed by the authors, a large positive value of *a* denotes a large time asymmetry. This index is further compared to what obtained with a large set of surrogate time series to obtain a *p*-value.
- *Ternary Coding*. Conceptually similar to several other tests here discussed, most notably the Permutation Patterns’ one, this test is based on symbolising the values of the time series, for then evaluating the difference in their frequency under a time-reversal operation [30]. Specifically, a time series x(t) is differentiated as d(t)=x(t+1)−x(t), and transformed according to:
  (3) $$s\left(t\right)=\left\{\begin{array}{cc} 1, & \mathrm{if}\;d(t)>\alpha \\ -1, & \mathrm{if}\;d(t)<-\alpha \\ 0, & \mathrm{otherwise}. \end{array}\right.$$
  Note that here α is calculated from the data distribution through its percentile; hence, a value of α=10 implies that the 10% largest values are encoded with a 1 symbol. The full test is performed by splitting the original time series into *D* non-overlapping segments, and by evaluating if the frequency of the 1 and −1 symbols is statistically different across them.
- *TP Length*. This test leverages the idea of trend patterns [65], i.e., sequences of consecutive values in the time series with a monotonous trend—either increasing or decreasing. Given a time series, this can be divided in trend patterns, and subsequently their length can be encoded in a probability distribution. As a final step, the length distribution of the original and time-reversed time series are compared, as these should be equal in a time reversible dynamics.
- *Visibility Graph*. Original irreversibility test [66] based on the analysis of the directed Horizontal Visibility Graph (dHVG) [50]—see the description for the local CC. In this case, a time series is classified as irreversible if the distributions of the number of links arriving at and departing from nodes (known, respectively, as the in- and out-degrees) are different in a statistically significant way, e.g., according to a Epps–Singleton test [67].
- *Zumbach*. In the original Ref. [68], Gilles Zumbach proposed several tests to detect time irreversibility in a financial time series; in spite of the very field-specific initial definition, they have been found useful outside their initial scope. The one considered here starts by transforming the original time series into a time series of returns, i.e., $\tilde{x}\left(t\right)=\log x\left(t\right)/x\left(t-1\right)$. Next, at each time *t*, two volatilities are calculated: a first one, called historical, with over all values between t−δt and *t*; and a second one, called realised, in the future data from t+γ (γ being called the granularity) to t+δt. The volatility is simply defined as the sum of the square of values in the considered time window. Note that, from the point of view of a value at time point *t*, these correspond to the past and future volatilities, and should therefore be equal under a time-reversal operation. Such equality, for all values of *t*, is finally tested using a two-sample Kolmogorov–Smirnov test.

**Table 1:** Synthesis of the irreversibility tests here considered, along with basic references and default parameters.

Test Name	Reference	Parameters
BDS	[40,41]	m=2, r=1.5
COP	[43]	D=3
Costa Index	[46]	-
DFK	[47]	n=3, L=3
Diks	[48]	m=6, τ=1
Local CC	[51]	-
MS Trends	[52]	δ=20, d=2
Permutation patterns	[56]	D=3
Pomeau	[1]	τ=1
Ramsey	[62]	k=1
Skewness	[63]	-
Ternaty Coding	[30]	α=10
TP Length	[65]	-
Visibility Graph	[66]	-
Zumbach	[68]	δt=20, γ=3

### 2.2. Downsampling Algorithms

Some of the previously mentioned tests have some form of multiscale capability already included in their definition, which involves performing the analysis using non-consecutive data points. Specifically, permutation patterns, Pomeau’s, and Ramsey tests include an embedding lag parameter (respectively called *D*, τ, and *k*). Others could be trivially adapted; for instance, Costa Index could be modified to detect increments as x(t)>x(t+τ), as opposed to x(t)>x(t+1). Still, many tests were not designed with multiscale analyses in mind.

In order to provide a homogeneous test bed for all of the above tests, we here opted to pre-process the time series, as is frequently performed in the literature. Hence, given a time series x(t) and a scale parameter τ, the former is transformed into a new one through two downsampling algorithms:

- 1:N downsampling: $\hat{x}\left(t\right)=x\left(\tau t\right)$, i.e., one every τ elements are retained.
- Average downsampling: $\hat{x}\left(t\right)=\sum_{j=\tau t}^{\tau (t+1)}x\left(j\right)/\tau$. As the name implies, the new values correspond to the average of non-overlapping windows of size τ of the original time series.

As in the case of irreversibility tests, the software implementation of these two downsampling methods corresponds to the one available in (Irreversibility Tests Library at https://gitlab.com/MZanin/irreversibilitytestslibrary (accessed on 20 December 2024)). As a last note, all time series used in what follows are constructed to have the same length irrespective of τ. Thus, unless otherwise specified, we here consider time series $\hat{x}\left(t\right)$ of length $10^{4}$, which have been obtained from raw time series x(t) of length $10^{4}\tau$. This is necessary, as the *p*-value obtained by any test changes as a function of the quantity of input data; therefore, the latter has to be kept constant throughout the different values of τ.

### 2.3. Evaluating Statistical Significance

In the analyses that follow, the previously described tests will be applied to time series generated with different dynamical systems, in order to evaluate whether they are time irreversible and the intensity of such irreversibility. The software implementation of the tests always yields a *p*-value—see the above description and the online documentation for details about how this is obtained. Given a set of parameters, including for instance a given value of τ, each test is applied to a large set of synthetic time series and the resulting *p*-values are synthesised in two metrics: (i) the fraction of time series whose *p*-value is smaller than a threshold $\alpha =10^{-3}$; and (ii) the median of the *p*-values.

The first metric is equivalent to what a practitioner would do when evaluating the statistical significance for a single time series, and assesses the probability of obtaining a significant value. Note that, in the ideal case and given a set of parameters, one would expect all generated time series to be either time reversible or irreversible; yet, due to the stochastic nature of the generation process, and in some cases of the tests themselves, intermediate situations are frequently found. On the other hand, the second metric measures the average intensity of the irreversibility, using the *p*-value as a proxy for it. While individual *p*-values are the result of a formal statistical test, the corresponding median is not to be understood as such, but only as a quantitative proxy of the intensity.

## 3. A Synthetic Model for Multiscale Irreversibility

**Figure 1:**
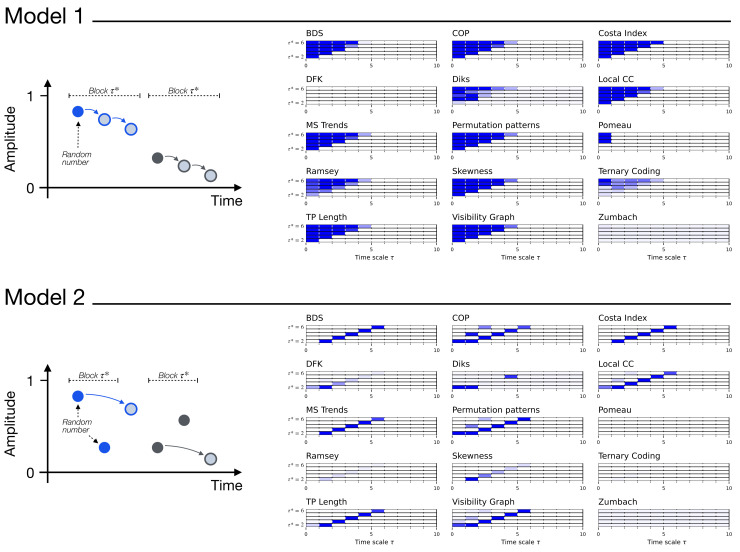
Synthetic models of multiscale irreversibility. The top and bottom parts correspond to the models described, respectively, in Equations (4) and (5). The left images graphically represent how the time series are created—see the main text for in-depth explanations; the detected irreversibility is reported in the right part. Specifically, each bar reports the evolution of the median *p*-value yielded by each test, as a function of the downsampling τ, as calculated over 200 independent realisations. Colour intensities indicate the *p*-value, from 1.0 (light shades, not irreversible) to 0.0 (dark shades, irreversible). From bottom to top, bars in each set correspond to different values of $\tau^{*}$, from 2 to 6.

In order to illustrate how irreversibility can emerge in a time series in a multiscale way, we here consider two simple discrete and univariate models. The first one is defined as follows:

(4) $$x\left(t\right)=\left\{\begin{array}{cc} U(0,1) & ift=k\tau^{*},\\ {}[x(t-1)-\mu ]\;\mathrm{mod}\;1 & otherwise, \end{array}\right.$$

with k∈N, $0<\mu <1/\tau^{*}$, and U(0,1) an independent random number is drawn from a uniform distribution between zero and one. As illustrated in the top left part of Figure 1, this model creates time series with a block structure of length $\tau^{*}$. Within each block, the first value (see solid circles) is drawn at random, while subsequent ones are equal to the previous value shifted by μ. In other words, $\tau^{*}$ defines the scale of the irreversibility through a reset process. Finally, note that the shifted (i.e., non-random) values are kept in the range [0,1] by means of the modulus operator.

It is simple to visualise how and when irreversibility emerges in this model. When $\tau^{*}=2$, one of every two values is random, with the intervening ones being a function of the previous points. If the raw time series is analysed, this is clearly irreversible, as x(t) is generally smaller than x(t−1) for even values of *t*. The time series is thus non-stationary, except for the hard resets introduced at fixed intervals. On the other hand, let us suppose that the time series is downsampled by a factor of two, i.e., one of every two points is retained. In this case, the resulting time series is either a set of random values (if even *t*s are retained), or a set of random values shifted by μ (if odd *t*s are retained): in both cases, the time series is completely stochastic and hence time-reversible. Let us next move to the case $\tau^{*}=3$. Similarly to the previous case, the raw time series are irreversible due to the trend introduced by μ; yet, here the time series created by discarding one every two points is also irreversible, as the trend will be visible for some *t*s—for instance, in the top example of Figure 1, the first and third points will be retained, hence creating an irreversibility. In short, this first model should be detected as irreversible only if $\tau <\tau^{*}$, and time-reversible otherwise.

An additional variation of this model is also illustrated in the bottom part of Figure 1, and is defined as follows:

(5) $$x\left(t\right)=\left\{\begin{array}{cc} U(0,1) & \mathrm{if}\;t\;\mathrm{mod}\;\left(\tau^{*}+1\right)<\tau^{*},\\ {}[x(t-\tau^{*})-\mu ]\;\mathrm{mod}\;1 & \mathrm{otherwise}. \end{array}\right.$$

In this case, blocks of $\tau^{*}$ random values are generated; next, a data point *t* is added, defined as the value at time $t-\tau^{*}$, further modified by μ. Let us again analyse what the result of an irreversibility evaluation should be. First of all, when considering the raw time series (i.e., τ=1), no irreversibility should be detected; note that, while one of every $\tau^{*}+1$ points is a function of a previous one, such a signal is very weak and is obfuscated by all the other random values. A trend is only included in the downsampled time series when the two corresponding points are sampled, i.e., the data points at time *t* and $t+\tau^{*}$. In other words, this second model should be detected as irreversible only when $\tau =\tau^{*}$, and time-reversible otherwise.

The right part of Figure 1 reports when each irreversibility test yield statistically significant results, for the two models of Equations (4) (top) and (5) (bottom); and as a function of the model time scale $\tau^{*}$, and the downsampling factor τ. In all cases, 200 time series of length $10^{4}$ were analysed, and with μ=0.05. Results are generally as expected, with most test detecting time irreversibility for $\tau <\tau^{*}$ in the former case, and for $\tau =\tau^{*}$ in the latter case. At the same time, some differences between tests start to emerge: to illustrate, the Ternary Coding test is more effective in the first case, while the DFK test detects irreversibility only in the second model. Also, Pomeau’s and Zumbach’s tests fail in both cases. These differences will further be explored in the next sections.

Beyond the differences between tests, these two synthetic models illustrate two complementary ways in which the multiscale nature of irreversibility can emerge. The first one, represented by the model of Equation (4), is through the loss of memory: the dynamical system is irreversible, but such a property depends on the memory of the past dynamics, which is lost due to the hard reset imposed at the time scale defined by $\tau^{*}$. The resulting time series are thus irreversible up to that time scale, but appear as stochastic above it. Some examples of this will include the geometric Brownian motion with stochastic resetting, presented in Section 6; and classical chaotic maps, see Section 7. The second one, represented by the model of Equation (5), consists of systems for which the irreversibility only manifests when one (or multiple) specific time scale(s) is (are) analysed, while the dynamics is time-reversible at all other scales. When analysing the evolution of the test significance as a function of τ, for instance through the evolution of the *p*-value, this will manifest as local minima. The clearest example of such behaviour will be seen in the next section, with the Lorenz dynamical system.

## 4. The Lorenz Dynamical System

We then move to the analysis of synthetic time series generated by the Lorenz dynamical system, which was chosen as it presents richer multiscale dynamics. For the sake of clarity, in what follows the main figures represent a synthetic version of the results, and specifically the evolution of the average *p*-value obtained by each test as a function of the time scale τ. This is represented through bars, with the colour intensity depicting the *p*-value—dark shades indicate more statistically significant results. At the same time, the complete results are reported in Supplementary Material, both in terms of the evolution of the *p*-value and of the fraction of the statistically significant tests as a function of τ.

The Lorenz dynamical system, introduced in 1963 as a minimal model for weather forecast [69], is one of the first systems known to exhibit a chaotic behaviour. It is defined as the following set of three differential equations:

$$\begin{array}{cc} \frac{dx}{dt} & =\sigma (y-x)\\ \frac{dy}{dt} & =x(\rho -z)-y\\ \frac{dz}{dt} & =xy-\beta z. \end{array}$$

We here consider the parameters σ=10, ρ=28, and β=2.667, thus in the chaotic regime; time series are generated by integrating with a time step of δt=0.01. In what follows we specifically focus on the evolution of *z*, although similar results can be obtained with the two other variables.

**Figure 2:**
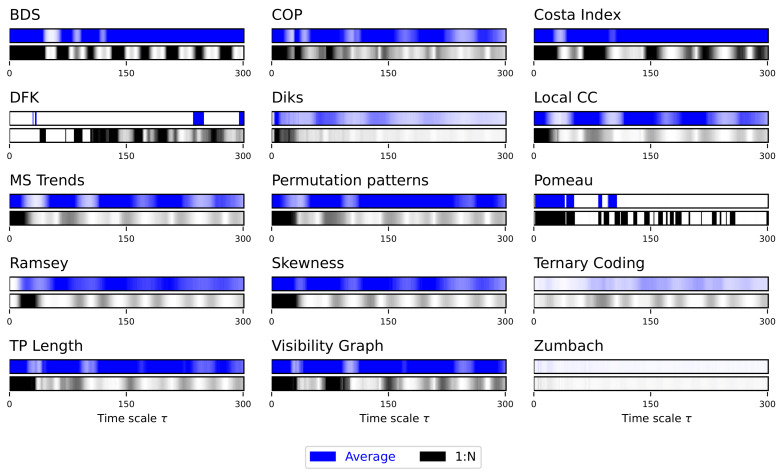
Evolution of the irreversibility for the Lorenz dynamical system. Each bar reports the evolution of the median *p*-value yielded by each test, as a function of the downsampling τ. Black bars correspond to a 1:N downsampling, blue bars to the average downsampling. Colour intensities indicate the *p*-value, from 1.0 (light shades, not irreversible) to 0.0 (dark shades, irreversible). Results correspond to 200 independent realisations. See Figures S1 and S2 for full results.

Due to the auto-correlation structure in the time series, the result is also a structure in the obtained irreversibility—note that this was already illustrated in previous works [52,56,70]. As previously explained, the main results are synthesised in Figure 2, while complete results are presented in Figures S1 and S2. The multiscale structure is clearly visible, as indicated by the dark bands inside each bar of Figure 2. At the same time, several interesting facts ought to be highlighted. First of all, the average downsampling (blue bars and lines) generally yield smaller *p*-values than the 1:N downsampling strategy—something that will be common, but not constant, throughout other time series types. Secondly, the multiscale structure can only be really appreciated when considering the evolution of the *p*-value, as e.g., in Figure 2 and Figure S1; conversely, measuring the fraction of irreversible time series yield almost always a 100%, irrespectively of τ, for the average downsampling, see Figure S2.

**Figure 3:**
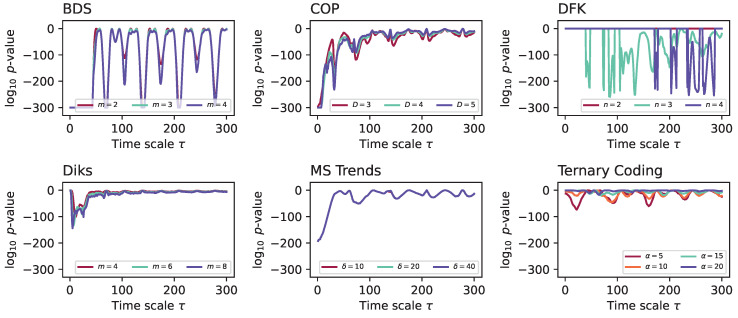
Evolution of the median *p*-value for the Lorenz dynamical system, when time series are downsampled using the 1:N approach, as a function of the main parameter of each test. Results correspond to 200 independent realisations.

Using the same dynamical system, we explore two additional characteristics of the considered irreversibility tests. Firstly, in Figure 3, we report the evolution of the median *p*-value yielded by a subset of the tests when varying their main parameters—see also Table 1 for default values. In most cases, varying these parameters has a minor impact on the results. The major exception is the DFK test, for which larger values of *n* (the number of partitions in which data values are grouped) seem to be needed to detect irreversibilities for large τs. The importance of correctly tuning the parameters of this test will also be confirmed below for other dynamical systems—see Figures S5 and S7.

**Figure 4:**
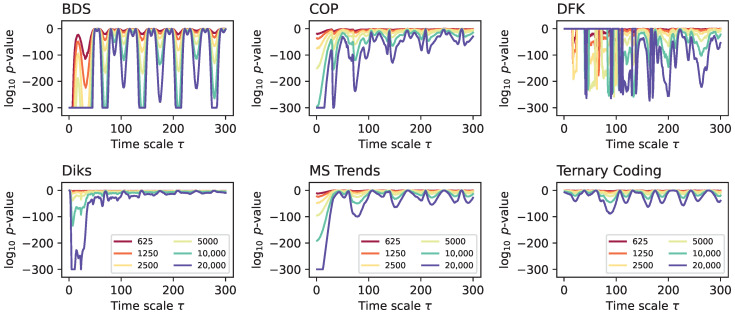
Evolution of the median *p*-value for the Lorenz dynamical system, when time series are downsampled using the 1:N approach, as a function of the length of the final (i.e., downsampled) time series (see colour code in legends). Results correspond to 200 independent realisations.

Secondly, Figure 4 reports the evolution of the median *p*-value yielded by the same six irreversibility tests, this time for different time series lengths. Here, the results are as expected, with longer time series being associated with smaller *p*-values in an almost linear fashion. Most importantly, the same multiscale structures appear across all time series length, indicating that this is not a defining aspect in the analysis.

**Figure 5:**
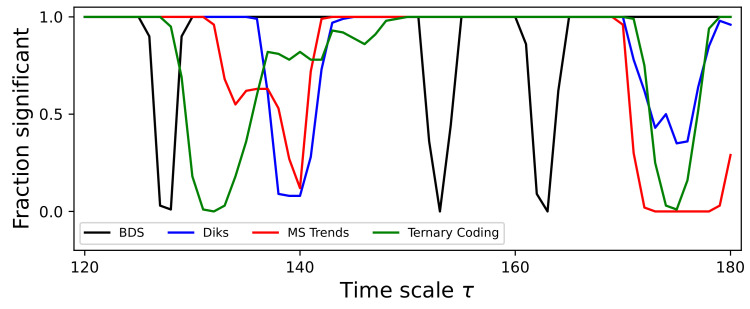
Zoom of the evolution of the irreversibility for the Lorenz dynamical system. The plot reports the evolution of the fraction of time series identified as irreversible in a statistically significant way (*p*-value $<10^{-3}$) by four tests (see legend), for 120≤τ≤180. Time series have been processed with the 1:N downsampling strategy, and thus correspond to the black lines of Figure S2. All results correspond to the median over 200 independent realisations.

As a final issue, it may be tempting to assume that all tests are detecting the same fine multiscale irreversibility structure, albeit with different intensities. A closer look nevertheless depicts a more complex situation. Specifically, Figure 5 shows a zoomed in fraction of the time series detected as significant for τ between 120 and 180, and focusing on four tests. It can be appreciated how all four tests display some minima for some values of τ, but that the specific value is seldom the same. For instance, Diks and MS Trends find a lack of irreversibility for τ=140, but not BDS (which finds it for τ<130) nor Ternary Coding (130<τ<140). Similarly, only BDS presents drops between 150 and 170; and detects all time series as irreversible for τ>170, as opposed to the other three tests. These results highlight the fact that the multiscale structure identified by a test does not necessarily correspond to the real multiscale structure of the system—a topic that will be discussed in Section 9.

## 5. The Asymmetric Weierstrass Function

We next move to the analysis of the asymmetric Weierstrass function; in spite of being less well-known than the Lorenz model, it presents the advantage of having specifically been created to generate time series with multiscale irreversibility [29]. The starting point is the classical Weierstrass function, an example of a continuous but nowhere differentiable function, defined as $W\left(x\right)=\sum_{n=0}^{\infty }a^{n}\cos\left(b^{n}\pi x\right)$ [71]. Note that, since this function is a composition of cosine waves, it is also perfectly time-reversible. This can be changed by substituting the cosine function with an asymmetric sawtooth as follows:

(6) $$W_{A}\left(x\right)=\sum_{n=0}^{n_{\max}}f^{-kH}S\left(2\pi f^{k}t;w\right).$$

Here, *f* is the minimum frequency, and *H* is the scaling (or Hurst) exponent (here set to 1). w∈[0,1] is the asymmetry parameter that determines the relative position of the maximum within the sawtooth function *S*; unless otherwise specified, we consider w=0.2. Additionally, $n_{\max}$ is the maximum number of harmonics included in the time series, here set to 50.

**Figure 6:**
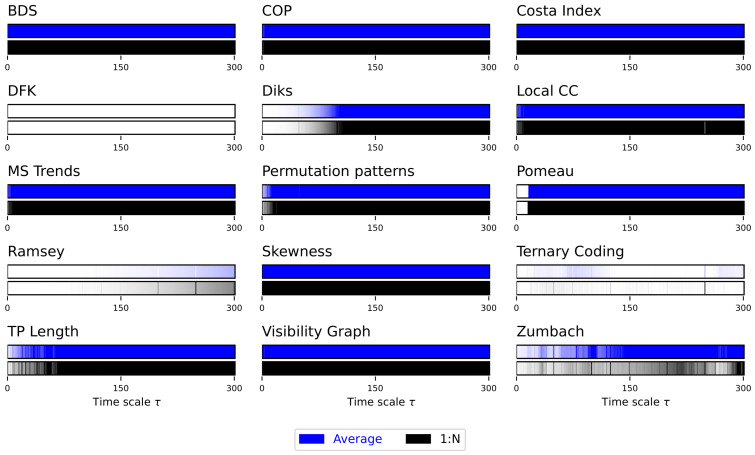
Evolution of the irreversibility for the asymmetric Weierstrass function. Each bar reports the evolution of the median *p*-value yielded by each test, as a function of the downsampling τ. Black bars correspond to a 1:N downsampling, blue bars to the average downsampling. Colour intensities indicate the *p*-value, from 1.0 (light shades, not irreversible) to 0.0 (dark shades, irreversible). See Figures S3 and S4 for full results. See also Figure S5 for results yielded by the DFK test for different parameters’ combinations.

The results presented in Figure 6, and further confirmed in Figures S3 and S4, suggest that the obtained time series are always irreversible, even though most tests struggle to detect such irreversibility for small values of τ. This is due to the the maximum number of harmonics included in the time series, i.e., $n_{\max}$, which limits the irreversibility content in very short time scales. Such gradual evolution to irreversibility is especially clear for some tests, e.g., Diks, Ramsey, TP Length, and Zumbach. Notably, these same tests present spikes in the evolution of the *p*-value, when the time scale τ matches the frequency of the sawtooth components—see Figure S3.

**Figure 7:**
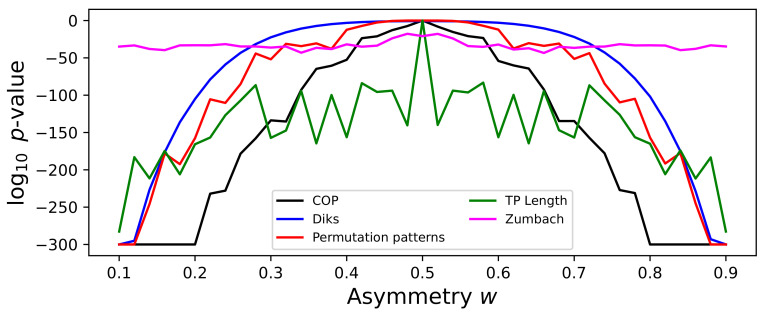
Evolution of the irreversibility as a function of the asymmetry parameter *w* of the asymmetric Weierstrass function. The plot reports the evolution of the median *p*-value yielded by five tests (see legend), τ=110. Time series have been processed with the 1:N downsampling strategy, and thus correspond to the black lines of Figure S4. All results correspond to the median over 200 independent realisations.

As previously discussed, the asymmetric Weierstrass function includes a parameter *w* defining the asymmetry of the sawtooth components, and hence the irreversibility of the time series. Figure 7 reports the evolution of the irreversibility, specifically of the median *p*-value, as detected by five tests. As expected, most of them present a bell-shaped evolution, with no asymmetry detected for w=0.5, which corresponds to a symmetric triangular wave. The specific shape, and hence the sensitivity to changes in *w*, is nevertheless not equal, with the COP test being the one requiring less variations in this parameter. It is also interesting to see the case of the Zumbach test, which still detects a minor irreversibility for w=0.5; this suggests that this test is also describing some other characteristics of the time series beyond time irreversibility.

**Figure 8:**
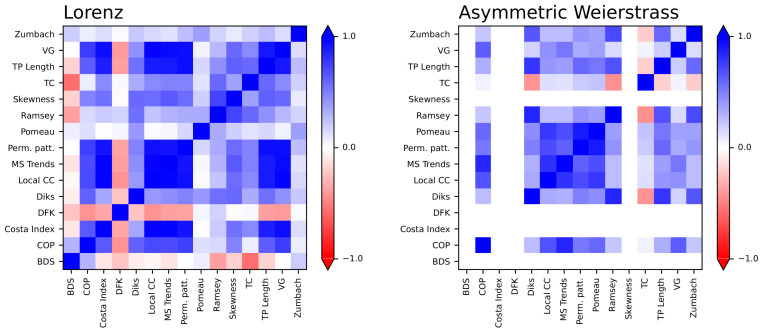
Correlation between tests. The two plots report the rank correlation coefficients between the *p*-value obtained by each test, for the Lorenz dynamical system (**left**) and the asymmetric Weierstrass function (**right**). *p*-values have been obtained using the 1:N downsampling, with τ ranging from 1 to 300. White rows and columns indicate cases in which the rank correlation could not be calculated, for instance for including *p*-values constant with respect to τ.

The natural question that may be asked at this stage is whether the 15 considered tests yield similar, or at least compatible, results across the 2 dynamical systems described up to here. In other words, are the tests that are most sensitive in the case of the Lorenz system also the most sensitive in the asymmetric Weierstrass one? This is an open problem in the assessment of irreversibility in real-world time series: due to the generality of the definition, different tests have been shown to perform heterogeneously in different problems [20]. The attentive reader will probably have already detected a similar scenario in the multiscale case here considered, with some tests behaving differently when analysing the two types of time series. To illustrate, the DFK and Ramsey tests detect irreversibility in the case of the Lorenz system (see Figure 2), but fail in the case of the asymmetric Weierstrass (see Figure 6); notably, the opposite behaviour can be seen for the Zumbach test. In order to better compare the 15 tests, Figure 8 reports the correlation between the *p*-values obtained by each of them as a function of τ, for the Lorenz (left panel) and asymmetric Weierstrass (right panel) dynamical systems. While positive correlations are generally observed, some exceptions stand out, as e.g., the cases of the DFK and the BDS tests for Lorenz time series.

## 6. Brownian Motion Models

### 6.1. Fractional Brownian Motion

The first type of Brownian motion here considered is a fractional Brownian motion (fBM), a generalization of the classic Brownian motion in which a parameter *H*, called the Hurst exponent, controls the increments’ correlation. Specifically, when H>0.5 (respectively, H<0.5), consecutive increments are positively (negatively) correlated; the classical, uncorrelated Brownian motion is recovered for H=0.5. We here use an implementation of the generation algorithm proposed by Davies and Harte [72].

**Figure 9:**
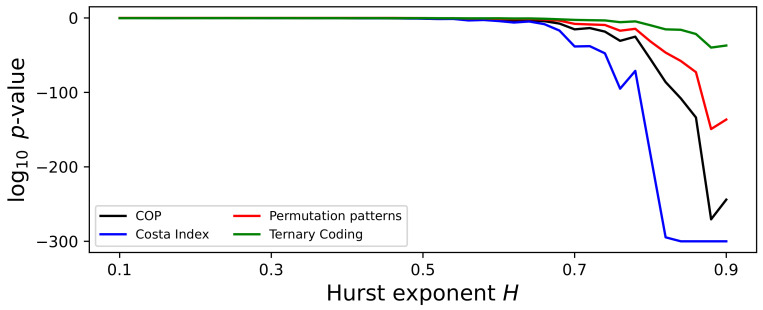
Evolution of the irreversibility, for time series generated by a fraction Brownian motion process, as a function of the Hurst exponent *H*. The plot reports the evolution of the median *p*-value yielded by four tests (see legend), with τ=10. Time series have been processed with the average downsampling strategy. All results correspond to the median over 200 independent realisations.

As a first point, it is interesting to discuss the irreversibility of a fBM process from a theoretical viewpoint. Note that this should not be confused with the associated process of the residuals, which is known as fractional Gaussian noise, a time-reversible correlated noise. A fBM process with Hurst exponent *H* has an expected value of zero, and its variance grows as $t^{2H}$; as such, it is not stationary, and consequently irreversible. At the same time, it has to be noted that the variance appears when several time series are considered at the same time, but that this aspect is more difficult to detect in a single realisation of the process. Still, time irreversibility also manifests in the persistent behaviour obtained for H>0.5, i.e., the time series either increase or decrease with time; this is another form of non-stationarity, and hence of irreversibility. These considerations make the detection of this property more challenging. To illustrate, Figure 9 reports the evolution of the $\log_{10}p$-value as a function of *H*, with τ=10; irreversibility is only detected when considering values of *H* above 0.7, and not across the full domain, as theoretically expected. In other words, even when considering single-scale versions of this property, tests can only rely on the presence of constant trends.

**Figure 10:**
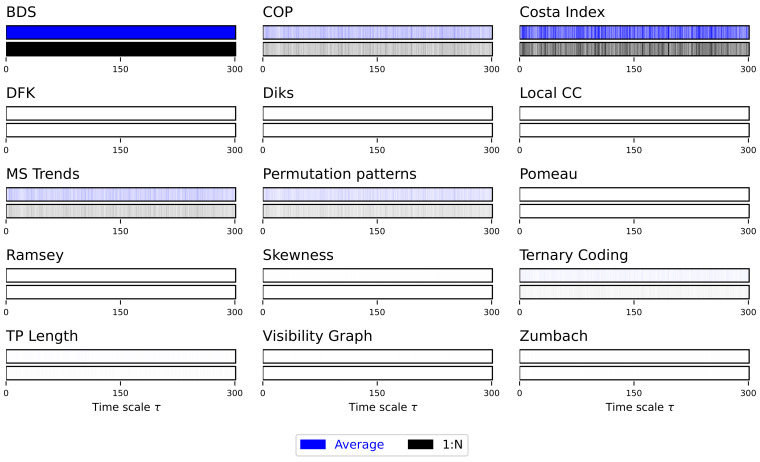
Evolution of the irreversibility for the fractional Brownian motion process with H=0.75. Each bar reports the evolution of the median *p*-value yielded by each test, as a function of the downsampling τ. Black bars correspond to a 1:N downsampling, blue bars to the average downsampling. Colour intensities indicate the *p*-value, from 1.0 (light shades, not irreversible) to 0.0 (dark shades, irreversible). See Figure S6 for full results. See also Figure S7 for results yielded by the DFK test for different parameters’ combinations.

In spite of these limitations, the results obtained by the different tests as a function of τ, for H=0.75, are quite interesting. As shown in Figure 10 (see also Figure S6 for details), even though only a few tests are able to detect the long-scale correlations in the data, the scale parameter does not affect the results. This is in line with what was expected, being the fBM a self-similar process; hence, its irreversibility is constant independently of the considered time scale.

### 6.2. Geometric Brownian Motion with Stochastic Resetting

As a second type, we consider the Geometric Brownian Motion model with stochastic resetting (srGBM for short). This model, recently proposed in [73], consists of a classical geometric Brownian motion in which the dynamics is randomly reset to the initial value, in order to better mimic the behaviour of real systems like financial markets and biological populations. Most importantly, while a standard GBM is non-stationary and hence irreversible, the presence of the stochastic reset tends to make the resulting time series both stationary [73] and time-reversible [74]. More in details, the dynamics is defined as follows:

(7) $$dx\left(t\right)=\left(1-Z_{t}\right)x\left(t\right)\left[\delta dt+\sigma dW\right]+Z_{t}\left[x_{0}-x\left(t\right)\right],\;x_{0}>0,$$

where x(t) is the evolution of the system with respect to time. The Wiener increment of the process dW is characterised with zero mean 〈dW〉=0 and correlation function $\langle dW_{t}dW_{s}\rangle =\delta \left(t-s\right)dt$. δ further denotes the drift amplitude, σ the standard deviation, and $x_{0}>0$ the initial value of x(t), $x\left(0\right)=x_{0}$. The resetting is introduced with a random variable $Z_{t}$ which takes the value 1 when a resetting to the initial position takes place, and 0 when there is no resetting. Note that the two aforementioned forces, i.e., the drift and the stochastic resetting, push the resulting time series, respectively, towards irreversibility and reversibility [74].

**Figure 11:**
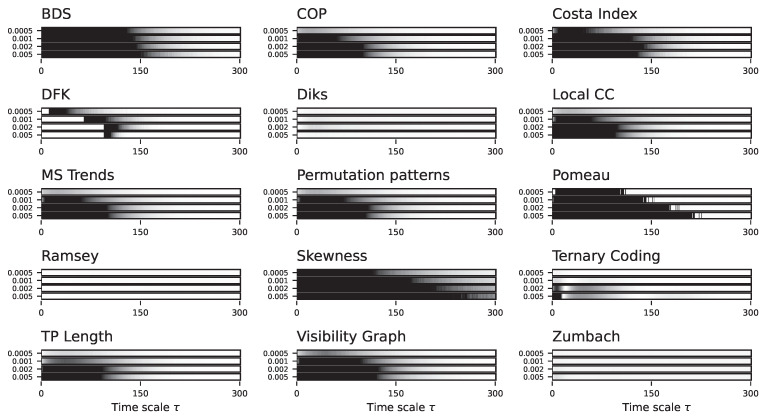
Evolution of the irreversibility for the srGBM dynamical system. Each bar reports the evolution of the median *p*-value yielded by each test, as a function of the downsampling τ. Groups of bars report results as a function of the drift, from $\delta =5\cdot 10^{-4}$ (top) to $\delta =5\cdot 10^{-3}$ (bottom). Colour intensities indicate the *p*-value, from 1.0 (light shades, not irreversible) to 0.0 (dark shades, irreversible). See Figure S8 for full results.

As can be appreciated in Figure 11, most tests detect a structure: the time series move from being irreversible for low values of τ, to time irreversible. When observing more in details the results in Figure S8, it can be seen that a maximal irreversibility is generally obtained in the range 1<τ<100. The threshold at which this transition occurs changes depending on the drift (see individual bars in each panel of Figure 11, and lines in Figure S8).

The reason for this multiscale irreversibility can be understood when considering the processes that make the time series reversible or irreversible. Initially, the original time series (i.e., for τ=1) are by construction time irreversible, due to the drift in the generative process; yet, such drift may be too small to be reliably detected by the tests. When introducing a small downsampling (well below the reset probability), the result is interpreted as a similarly constructed time series, but with a drift increased by a factor τ; consequently, the irreversible nature of the dynamics is more easily described. Finally, for large values of τ, the resetting mechanism dominates over the Brownian motion dynamics, yielding time series akin to random ones—and hence time reversible.

Most importantly, these results suggest that an observed increase in irreversibility (through, for instance, a decrease in the *p*-value) does not necessarily imply a change in the underlying dynamics of the system. In this case, the irreversibility is the same for τ=1 and larger values; tests have nevertheless less problems in detecting such properties in the latter case.

## 7. Chaotic Discrete Maps

As a final point, we explore the multiscale irreversibility of two classical chaotic and dissipative (hence, time irreversible) maps. The first is the logistic map, defined as $x_{n+1}=rx_{n}\left(1-x_{n}\right)$; it displays a chaotic dynamic for r=4, with Lyapunov exponent λ=ln2. Next, the Henon map, a bidimensional map defined as $x_{n+1}=1-ax_{n}^{2}+y_{n}$, $y_{n+1}=bx_{n}1$. We consider only the time series generated by the *x* variable, in the chaotic window (largest Lyapunov exponent λ≈0.419) defined by a=1.4 and b=0.3.

**Figure 12:**
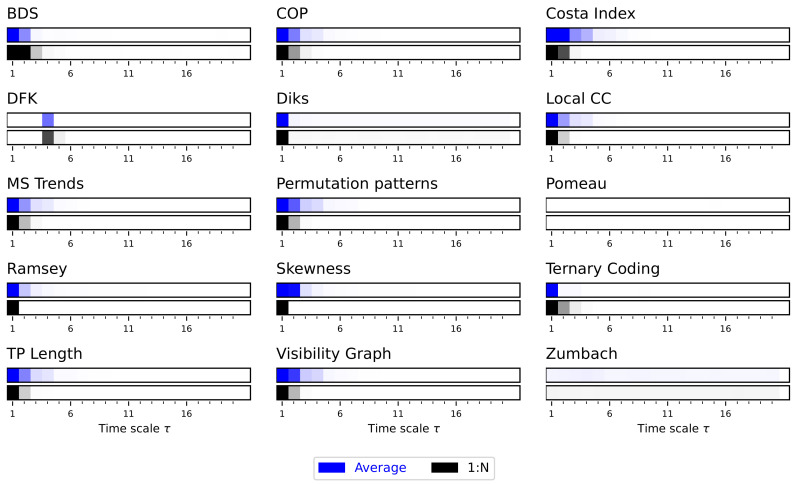
Evolution of the irreversibility for the Logistic map. Each bar reports the evolution of the median *p*-value yielded by each test, as a function of the downsampling τ. Black bars correspond to a 1:N downsampling, blue bars to the average downsampling. Colour intensities indicate the *p*-value, from 1.0 (light shades, not irreversible) to 0.0 (dark shades, irreversible). See Figures S9 and S10 for full results.

**Figure 13:**
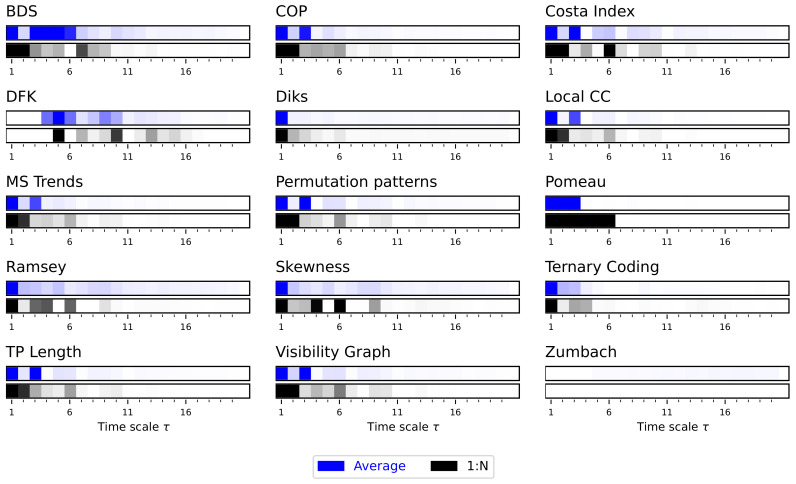
Evolution of the irreversibility for the Henon map. Each bar reports the evolution of the median *p*-value yielded by each test, as a function of the downsampling τ. Black bars correspond to a 1:N downsampling, blue bars to the average downsampling. Colour intensities indicate the *p*-value, from 1.0 (light shades, not irreversible) to 0.0 (dark shades, irreversible).

The motivation for studying these maps is that, given their chaotic nature, their predictability is lost in a time scale proportional to the inverse of the Lyapunov exponent (also called the Lyapunov time); hence, for τ>1/λ, the time series by them generated become effectively random and therefore time-reversible. This is confirmed by the results presented in Figure 12 and Figure 13, for, respectively, the logistic and Henon maps. Note how irreversibility is almost lost for λ>2 in the former, and for λ>3 in the latter, as theoretically expected.

## 8. Real-World Data: Bitcoin Price Evolution

Before moving to the discussion of the main results here obtained, we complement what previously reported with an analysis of the multiscale irreversibility of a real-world system. We specifically consider the evolution of Bitcoin prices; this financial asset presents several interesting characteristics that deviate from those of more traditional instruments, including a high volatility [75,76] and lower efficiency [77,78]. Data for the Bitcoin–US Dollar pair have been obtained from (CryptoDataDownload at https://www.cryptodatadownload.com/data/gemini/, accessed on 12 January 2025), and include the evolution of closing prices with a 1-minute time resolution between years 2015 and 2024, both included. As in previous cases, the length of time series has been kept constant irrespective of the value of τ; hence, the analyses below are based on randomly drawn subsets of the time series, of length $10^{3}\tau$.

**Figure 14:**
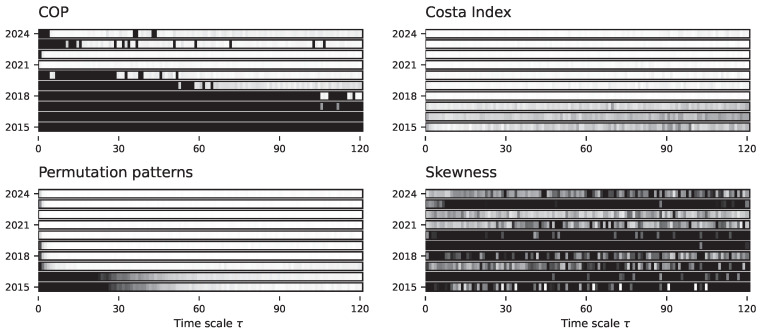
Evolution of the irreversibility in the Bitcoin price data. Each bar reports the evolution of the median *p*-value yielded by four tests (see name of top of panels), as a function of the downsampling τ. Additionally, each bar depicts the result for one year of data, from bottom (2015) to top (2024). Colour intensities indicate the *p*-value, from 1.0 (light shades, not irreversible) to 0.0 (dark shades, irreversible). Results for all tests are reported in Figure S11.

Figure 14 reports the evolution of the irreversibility as a function of the time scale τ, for the four tests yielding the most interesting results; full details are also included in Figure S11. Results are reported by years, from 2015 (bottom bar of each panel) to 2024 (top bar), as the Bitcoin market has undergone a significant evolution in its capitalisation—as also previously seen in studies focusing on its single scale time irreversibility, see Refs. [65,79,80]. A rich multiscale structure, and its evolution, can be observed. Specifically, three out of four tests indicate that Bitcoin prices were irreversible at all time scales in their initial years. More recently, this has evolved into an irreversibility only in short time scales (see COP and Permutation patters), or to a weaker irreversibility (Costa index and Skewness). This is aligned with the idea that the Bitcoin market has been increasing its capitalisation, resulting in a more efficient and random (hence, less time-reversible) dynamics. On the other hand, as partial reversal of this dynamics can be observed for year 2023, with a minor increase in the irreversibility; yet, except for the introduction of Non-Fungible Tokens (NFTs) in the blockchain [81], nothing major happened that year to explain such behaviour.

## 9. Discussion and Conclusions

We evaluated the ability of some of the main algorithmic procedures to detect the multiscale time irreversibility of several well-known synthetic dynamical systems. Most tests yielded a somehow comparable picture of the general evolution of irreversibility across scales. This was especially true for BDS, COP, Costa Index, Permutation Patterns, Skewness, TP Length and Visibility Graph. Other tests, especially Zumbach, DFK, and Ternary Coding, are more context-dependent, i.e., they may or may not detect irreversibility. In particular, most methods turn out to be able to identify systems that are, respectively, irreversible up to some scale, at some scales or at all scales, the latter case reflecting self-similarity of the underlying process. In some cases, irreversibility is detected only above a given temporal scale; this is related to the fact that some tests cannot incorporate information over long time scales, and therefore they only analyse local noise. Tests struggle with fBM, as they tend to only focus on local patterns of the time series. Hence irreversibility was detected only for large enough values of *H*, for which the self-similar nature of the process was correctly identified.

On the other hand, different tests yielded profoundly different results in terms of irreversibility’s finer structure. This point is illustrated in Figure 5, where minima appear for different τ depending on the test, leading to an apparent negative correlation between them, see Figure 8. This result suggests that while often used for the same purpose of detecting time-reversal symmetry, these methods constitute filters highlighting at least partially different properties of the phenomenon generating a given time series. In particular, these methods may differ in terms of sensitivity to particular signal properties, such as nonlinearity, or to the way the system is coarse-grained.

More generally, the differences in time scales yielded by different measures invite a discussion on the issue of time scales, their significance, and the way the chosen method of data analysis may affect their estimation. Any method designed to detect time series’ irreversibility could in principle reveal intrinsic time scales of the underlying process and, as a result, allow reconstructing the temporal structure of nonequilibrium system’s activity. Coarse-graining procedures are designed to lower the resolution in order to reduce in an “acceptable” way the information contained in the system. This involves transformations of the way a system is described at various scales, which involve in a way or another downsampling. While incomplete information and coarse-graining affect entropy production estimates, the relative entropy associated with a finite coarse-grained continuous trajectory provides reasonably accurate lower bounds for the dissipation, even with only a small number of measurement points [82,83,84,85]. For instance, it has been conjectured that the relative entropy obtained from *n* measurements approaches the exact value of the dissipation as $1/n^{2}$ for *n* large [83]. On the other hand, each method of data analysis comes with its own set of characteristic scales. The effective time scales of the system ultimately coincide with those allowed by the methods of data analysis, which in turn should neither introduce spurious scales nor conceal genuine ones [9]. For instance, the particular downsampling procedure affected estimates of time-reversal symmetry and its time scales. On this topic, our results suggest that downsampling by averaging usually provides better results than the 1:N strategy in terms of lower *p*-values. Note that this may sometimes be a drawback, as e.g., a fine multiscale structure can be more visible when using the 1:N approach, as there is less saturation.

Altogether, our results suggest that while various algorithms can detect irreversibility at various time scales, care should be taken in choosing the particular procedure, including the specific algorithm and the coarse-graining procedure. Whenever information about the analysed system’s dynamics is available, this ought to be taken into account, possibly through the use of tailored (and potentially simpler) irreversibility tests [46,68,86]. In short, care is required in the evaluation of the results, particularly in terms of intrinsic time scales of the underlying system.

## Data Availability

Data are contained within the article.

## Supplementary Materials

The following supporting information can be downloaded at: https://www.mdpi.com/article/10.3390/e27020126/s1, Figure S1: Evolution of the irreversibility for the Lorenz dynamical system (*p*-value); Figure S2: Evolution of the irreversibility for the Lorenz dynamical system (fraction); Figure S3: Evolution of the irreversibility for the asymmetric Weierstrass dynamical system (*p*-value); Figure S4: Evolution of the irreversibility for the asymmetric Weierstrass dynamical system (fraction); Figure S5: Evolution of the irreversibility for the fBM (fraction); Figure S6: Evolution of the irreversibility for the srGBM dynamical system (*p*-value); Figure S7: Evolution of the irreversibility for the Logistic map (*p*-value); Figure S8: Evolution of the irreversibility for the Logistic map (fraction); Figure S9: Evolution of the irreversibility in the Bitcoin price data.

## Abbreviations

The following abbreviations are used in this manuscript:

BDS	Brock, Dechert, and Scheinkman
CC	Clustering Coefficient
COPs	Continuous Ordinal Patterns
DFK	Daw, Finney and Kennel
dHVG	Directed Horizontal Visibility Graph
fBM	Fractional Brownian Motion
MS Trends	Micro-scale Trends
O.-U.	Ornstein–Uhlenbeck
srGBM	Geometric Brownian Motion with stochastic resetting
TP Length	Trend Patterns Length

## References

[1] Pomeau Y. (1982). Symétrie des fluctuations dans le renversement du temps. J. De Phys..

[2] Gnesotto F.S., Mura F., Gladrow J., Broedersz C.P. (2018). Broken detailed balance and non-equilibrium dynamics in living systems: A review. Rep. Prog. Phys..

[3] Needleman D., Dogic Z. (2017). Active matter at the interface between materials science and cell biology. Nat. Rev. Mater..

[4] Gaspard P. (2004). Time-reversed dynamical entropy and irreversibility in Markovian random processes. J. Stat. Phys..

[5] Parrondo J.M., Van den Broeck C., Kawai R. (2009). Entropy production and the arrow of time. New J. Phys..

[6] Gaspard P. (2005). Brownian motion, dynamical randomness and irreversibility. New J. Phys..

[7] Fodor É., Nardini C., Cates M.E., Tailleur J., Visco P., Van Wijland F. (2016). How far from equilibrium is active matter?. Phys. Rev. Lett..

[8] Kubo R. (1966). The fluctuation-dissipation theorem. Rep. Prog. Phys..

[9] Papo D. (2013). Time scales in cognitive neuroscience. Front. Physiol..

[10] Papo D. (2014). Measuring brain temperature without a thermometer. Front. Physiol..

[11] Sarracino A., Arviv O., Shriki O., De Arcangelis L. (2020). Predicting brain evoked response to external stimuli from temporal correlations of spontaneous activity. Phys. Rev. Res..

[12] Lindner B. (2022). Fluctuation-dissipation relations for spiking neurons. Phys. Rev. Lett..

[13] Deco G., Lynn C.W., Sanz Perl Y., Kringelbach M.L. (2023). Violations of the fluctuation-dissipation theorem reveal distinct nonequilibrium dynamics of brain states. Phys. Rev. E.

[14] Battle C., Broedersz C.P., Fakhri N., Geyer V.F., Howard J., Schmidt C.F., MacKintosh F.C. (2016). Broken detailed balance at mesoscopic scales in active biological systems. Science.

[15] Martínez I.A., Bisker G., Horowitz J.M., Parrondo J.M. (2019). Inferring broken detailed balance in the absence of observable currents. Nat. Commun..

[16] Lynn C.W., Cornblath E.J., Papadopoulos L., Bertolero M.A., Bassett D.S. (2021). Broken detailed balance and entropy production in the human brain. Proc. Natl. Acad. Sci. USA.

[17] Weiss G. (1975). Time-reversibility of linear stochastic processes. J. Appl. Probab..

[18] Lawrance A. (1991). Directionality and reversibility in time series. Int. Stat. Rev. Int. Stat..

[19] Stone L., Landan G., May R.M. (1996). Detecting time’s arrow: A method for identifying nonlinearity and deterministic chaos in time-series data. Proc. R. Soc. Lond. Ser. B Biol. Sci..

[20] Zanin M., Papo D. (2021). Algorithmic approaches for assessing irreversibility in time series: Review and comparison. Entropy.

[21] Puglisi A., Villamaina D. (2009). Irreversible effects of memory. Europhys. Lett..

[22] Gallavotti G. (2007). Fluctuation relation, fluctuation theorem, thermostats and entropy creation in nonequilibrium statistical physics. Comptes Rendus Phys..

[23] Cugliandolo L.F., Dean D.S., Kurchan J. (1997). Fluctuation-dissipation theorems and entropy production in relaxational systems. Phys. Rev. Lett..

[24] Egolf D.A. (2000). Equilibrium regained: From nonequilibrium chaos to statistical mechanics. Science.

[25] Tan T.H., Watson G.A., Chao Y.C., Li J., Gingrich T.R., Horowitz J.M., Fakhri N. (2021). Scale-dependent irreversibility in living matter. arXiv.

[26] Bernardi D., Shannahoff-Khalsa D., Sale J., Wright J.A., Fadiga L., Papo D. (2023). The time scales of irreversibility in spontaneous brain activity are altered in obsessive compulsive disorder. Front. Psychiatry.

[27] Zanin M., Güntekin B., Aktürk T., Hanoğlu L., Papo D. (2020). Time irreversibility of resting-state activity in the healthy brain and pathology. Front. Physiol..

[28] Costa M.D., Peng C.K., Goldberger A.L. (2008). Multiscale analysis of heart rate dynamics: Entropy and time irreversibility measures. Cardiovasc. Eng..

[29] Burykin A., Costa M.D., Peng C.K., Goldberger A.L., Buchman T.G. (2011). Generating signals with multiscale time irreversibility: The asymmetric weierstrass function. Complexity.

[30] Cammarota C., Rogora E. (2007). Time reversal, symbolic series and irreversibility of human heartbeat. Chaos Solitons Fractals.

[31] Hou F., Zhuang J., Bian C., Tong T., Chen Y., Yin J., Qiu X., Ning X. (2010). Analysis of heartbeat asymmetry based on multi-scale time irreversibility test. Phys. A Stat. Mech. Its Appl..

[32] Hou F.Z., Ning X.B., Zhuang J.J., Huang X.L., Fu M.J., Bian C.H. (2011). High-dimensional time irreversibility analysis of human interbeat intervals. Med Eng. Phys..

[33] Chladekova L., Czippelova B., Turianikova Z., Tonhajzerova I., Calkovska A., Baumert M., Javorka M. (2012). Multiscale time irreversibility of heart rate and blood pressure variability during orthostasis. Physiol. Meas..

[34] Wu X., Yang Q., Li J., Hou F. (2021). Investigation on the Prediction of Cardiovascular Events Based on Multi-Scale Time Irreversibility Analysis. Symmetry.

[35] Xia J., Shang P., Wang J., Shi W. (2014). Classifying of financial time series based on multiscale entropy and multiscale time irreversibility. Phys. A Stat. Mech. Its Appl..

[36] Xu M., Shang P. (2018). Multiscale time irreversibility analysis of financial time series based on segmentation. Nonlinear Dyn..

[37] Xie B., Kong D., Kong L., Kong W., Li L. (2018). Analysis of vertical upward oil-gas-water three-phase flow based on multi-scale time irreversibility. Flow Meas. Instrum..

[38] Iacobello G., Chowdhuri S., Ridolfi L., Rondoni L., Scarsoglio S. (2023). Coherent structures at the origin of time irreversibility in wall turbulence. Commun. Phys..

[39] Huang Y., Yang D., Wang L., Wang K. (2020). Classifying of welding time series based on multi-scale time irreversibility analysis and extreme learning machine. Chaos Solitons Fractals.

[40] Brock W.A., Hsieh D.A., LeBaron B.D. (1991). Nonlinear Dynamics, Chaos, and Instability: Statistical Theory and Economic Evidence.

[41] Rothman P. (1992). The comparative power of the TR test against simple threshold models. J. Appl. Econom..

[42] Brock W.A., Scheinkman J.A., Dechert W.D., LeBaron B. (1996). A test for independence based on the correlation dimension. Econom. Rev..

[43] Zanin M. (2023). Continuous ordinal patterns: Creating a bridge between ordinal analysis and deep learning. Chaos Interdiscip. J. Nonlinear Sci..

[44] Azami H., Escudero J. (2016). Amplitude-aware permutation entropy: Illustration in spike detection and signal segmentation. Comput. Methods Programs Biomed..

[45] Cuesta Frau D. (2019). Permutation entropy: Influence of amplitude information on time series classification performance. Math. Biosci. Eng..

[46] Costa M., Goldberger A.L., Peng C.K. (2005). Broken asymmetry of the human heartbeat: Loss of time irreversibility in aging and disease. Phys. Rev. Lett..

[47] Daw C., Finney C., Kennel M. (2000). Symbolic approach for measuring temporal “irreversibility”. Phys. Rev. E.

[48] Diks C., Van Houwelingen J., Takens F., DeGoede J. (1995). Reversibility as a criterion for discriminating time series. Phys. Lett. A.

[49] Strogatz S.H. (2001). Exploring complex networks. Nature.

[50] Lacasa L., Luque B., Ballesteros F., Luque J., Nuno J.C. (2008). From time series to complex networks: The visibility graph. Proc. Natl. Acad. Sci. USA.

[51] Donges J.F., Donner R.V., Kurths J. (2013). Testing time series irreversibility using complex network methods. Europhys. Lett..

[52] Zanin M. (2021). Assessing time series irreversibility through micro-scale trends. Chaos Interdiscip. J. Nonlinear Sci..

[53] Bandt C., Pompe B. (2002). Permutation entropy: A natural complexity measure for time series. Phys. Rev. Lett..

[54] Zanin M., Zunino L., Rosso O.A., Papo D. (2012). Permutation entropy and its main biomedical and econophysics applications: A review. Entropy.

[55] Leyva I., Martínez J.H., Masoller C., Rosso O.A., Zanin M. (2022). 20 years of ordinal patterns: Perspectives and challenges. Europhys. Lett..

[56] Zanin M., Rodríguez-González A., Menasalvas Ruiz E., Papo D. (2018). Assessing time series reversibility through permutation patterns. Entropy.

[57] Martínez J.H., Herrera-Diestra J.L., Chavez M. (2018). Detection of time reversibility in time series by ordinal patterns analysis. Chaos Interdiscip. J. Nonlinear Sci..

[58] Yao W., Yao W., Wang J., Dai J. (2019). Quantifying time irreversibility using probabilistic differences between symmetric permutations. Phys. Lett. A.

[59] Li J., Shang P., Zhang X. (2019). Time series irreversibility analysis using Jensen–Shannon divergence calculated by permutation pattern. Nonlinear Dyn..

[60] Zunino L., Olivares F., Ribeiro H.V., Rosso O.A. (2022). Permutation Jensen-Shannon distance: A versatile and fast symbolic tool for complex time-series analysis. Phys. Rev. E.

[61] Cox D.R., Gudmundsson G., Lindgren G., Bondesson L., Harsaae E., Laake P., Juselius K., Lauritzen S.L. (1981). Statistical analysis of time series: Some recent developments [with discussion and reply]. Scand. J. Stat..

[62] Ramsey J.B., Rothman P. (1996). Time irreversibility and business cycle asymmetry. J. Money Credit Bank..

[63] Koutsoyiannis D. (2019). Time’s arrow in stochastic characterization and simulation of atmospheric and hydrological processes. Hydrol. Sci. J..

[64] Vavoulogiannis S., Iliopoulou T., Dimitriadis P., Koutsoyiannis D. (2021). Multiscale temporal irreversibility of streamflow and its stochastic modelling. Hydrology.

[65] Morales Herrera J., Salgado-García R. (2024). Measuring irreversibility via trend pattern lengths. AIP Adv..

[66] Lacasa L., Nunez A., Roldán É., Parrondo J.M., Luque B. (2012). Time series irreversibility: A visibility graph approach. Eur. Phys. J. B.

[67] Epps T., Singleton K.J. (1986). An omnibus test for the two-sample problem using the empirical characteristic function. J. Stat. Comput. Simul..

[68] Zumbach G. (2009). Time reversal invariance in finance. Quant. Financ..

[69] Lorenz E.N. (1963). Deterministic nonperiodic flow. J. Atmos. Sci..

[70] Cocciaglia N., Lucente D. (2024). Detecting time-irreversibility in multiscale systems: Correlation and response functions in the Lorenz96 model. arXiv.

[71] Weierstrass K. (2019). On continuous functions of a real argument that do not have a well-defined differential quotient. Classics on Fractals.

[72] Davies R.B., Harte D.S. (1987). Tests for Hurst effect. Biometrika.

[73] Stojkoski V., Sandev T., Kocarev L., Pal A. (2021). Geometric Brownian motion under stochastic resetting: A stationary yet nonergodic process. Phys. Rev. E.

[74] Zanin M., Trajanovski P., Jolakoski P., Sandev T., Kocarev L. (2024). Evaluating Time Irreversibility Tests Using Geometric Brownian Motions with Stochastic Resetting. Symmetry.

[75] Katsiampa P. (2017). Volatility estimation for Bitcoin: A comparison of GARCH models. Econ. Lett..

[76] Klein T., Thu H.P., Walther T. (2018). Bitcoin is not the New Gold–A comparison of volatility, correlation, and portfolio performance. Int. Rev. Financ. Anal..

[77] Jakub B. (2015). Does Bitcoin follow the hypothesis of efficient market. Int. J. Econ. Sci..

[78] Sensoy A. (2019). The inefficiency of Bitcoin revisited: A high-frequency analysis with alternative currencies. Financ. Res. Lett..

[79] Martínez J.H., Ramasco J.J., Zanin M. (2023). On the complementarity of ordinal patterns-based entropy and time asymmetry metrics. Chaos Interdiscip. J. Nonlinear Sci..

[80] Herrera J.M., Salgado-García R. (2023). Trend patterns statistics for assessing irreversibility in cryptocurrencies: Time-asymmetry versus inefficiency. arXiv.

[81] Ante L. (2022). The non-fungible token (NFT) market and its relationship with Bitcoin and Ethereum. FinTech.

[82] Seifert U. (2019). From stochastic thermodynamics to thermodynamic inference. Annu. Rev. Condens. Matter Phys..

[83] Gomez-Marin A., Parrondo J.M., Van den Broeck C. (2008). Lower bounds on dissipation upon coarse graining. Phys. Rev. E—Stat. Nonlinear Soft Matter Phys..

[84] Esposito M. (2012). Stochastic thermodynamics under coarse graining. Phys. Rev. E—Sta. Nonlinear Soft Matter Phys..

[85] Lucente D., Baldassarri A., Puglisi A., Vulpiani A., Viale M. (2022). Inference of time irreversibility from incomplete information: Linear systems and its pitfalls. Phys. Rev. Res..

[86] Guzik P., Piskorski J., Krauze T., Wykretowicz A., Wysocki H. (2006). Heart rate asymmetry by Poincaré plots of RR intervals. Biomed. Eng./Biomed. Tech..

