# Supplementary material for: Algorithmic Approaches for Assessing Multiscale Irreversibility in Time Series: Review and Comparison
Source: Entropy (Basel). 2025 Jan 25;27(2):126. doi: 10.3390/e27020126 (PMC11854910; doi:10.3390/e27020126)
Supplement: Supplementary file 1 [file entropy-27-00126-s001.zip › entropy-3428993-supplementary.pdf]

Supplementary Material for:

Algorithmic approaches for assessing multiscale irreversibility in time series: Review and comparison

Massimiliano Zanin[1] David Papo[2,3]

[1] Instituto de Física Interdisciplinar y Sistemas Complejos IFISC (CSIC-UIB), Campus UIB, 07122 Palma de Mallorca, Spain

[2] Department of Neuroscience and Rehabilitation, Section of Physiology, University of Ferrara, Ferrara (Italy)

[3] Center for Translational Neurophysiology, Fondazione Istituto Italiano di Tecnologia, Ferrara (Italy)

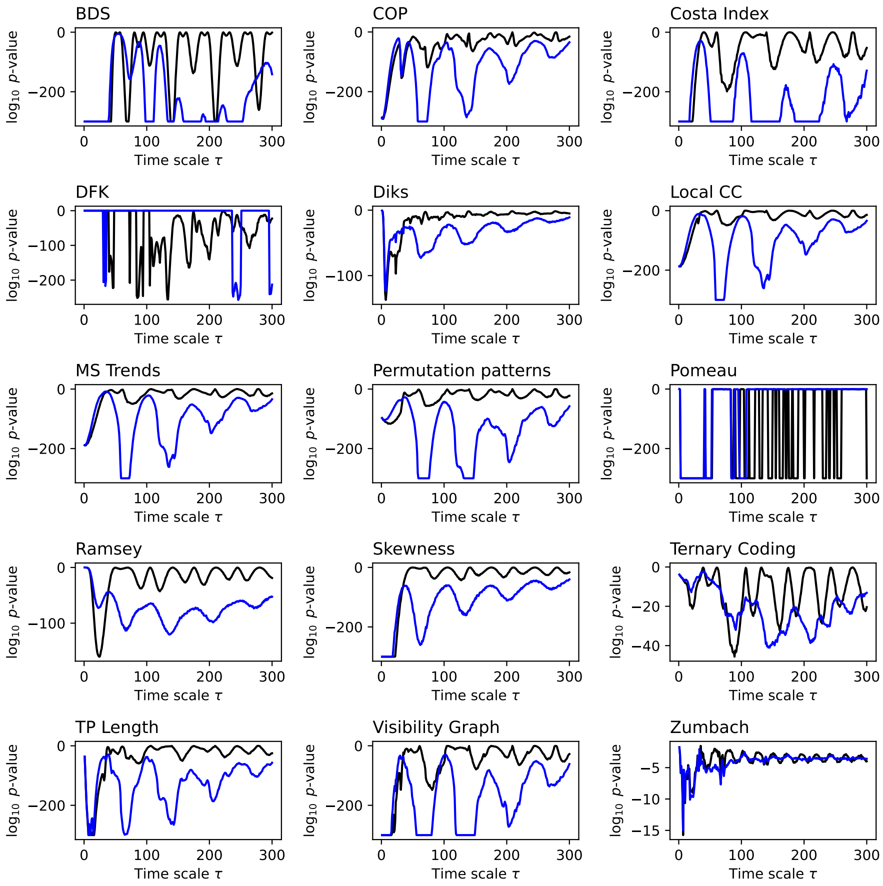

Figure S1: Evolution of the irreversibility for the Lorenz dynamical system (p-value). Each panel reports the evolution of the $\log_{10}$ of the p-value yielded by each test, as a function of the downsampling τ. Black lines correspond to a 1:N downsampling, blue lines to the average downsampling. p-values below 10^{-300} are set to that value. All results correspond to the median over 200 independent realisations.

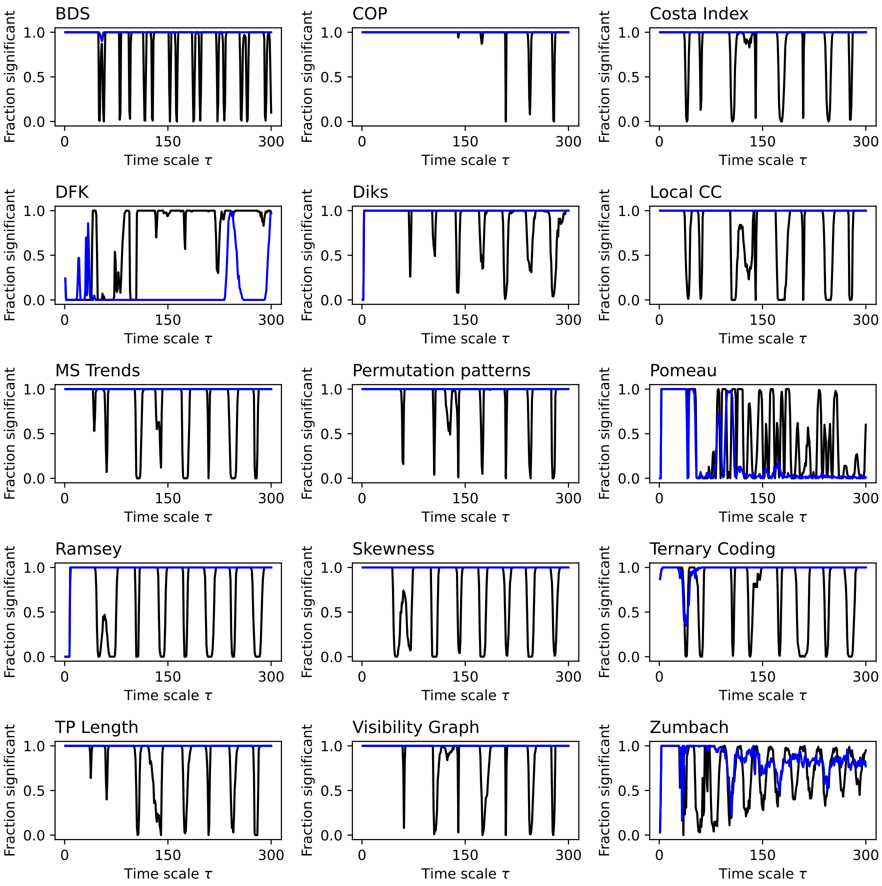

Figure S2: Evolution of the irreversibility for the Lorenz dynamical system (fraction). Each panel reports the evolution of the fraction of time series identified as irreversible in a statistically significant way (p-value $< 10^{-3}$) by each test, as a function of the downsampling τ. Black lines correspond to a 1:N downsampling, blue lines to the average downsampling. All results correspond to the median over 200 independent realisations.

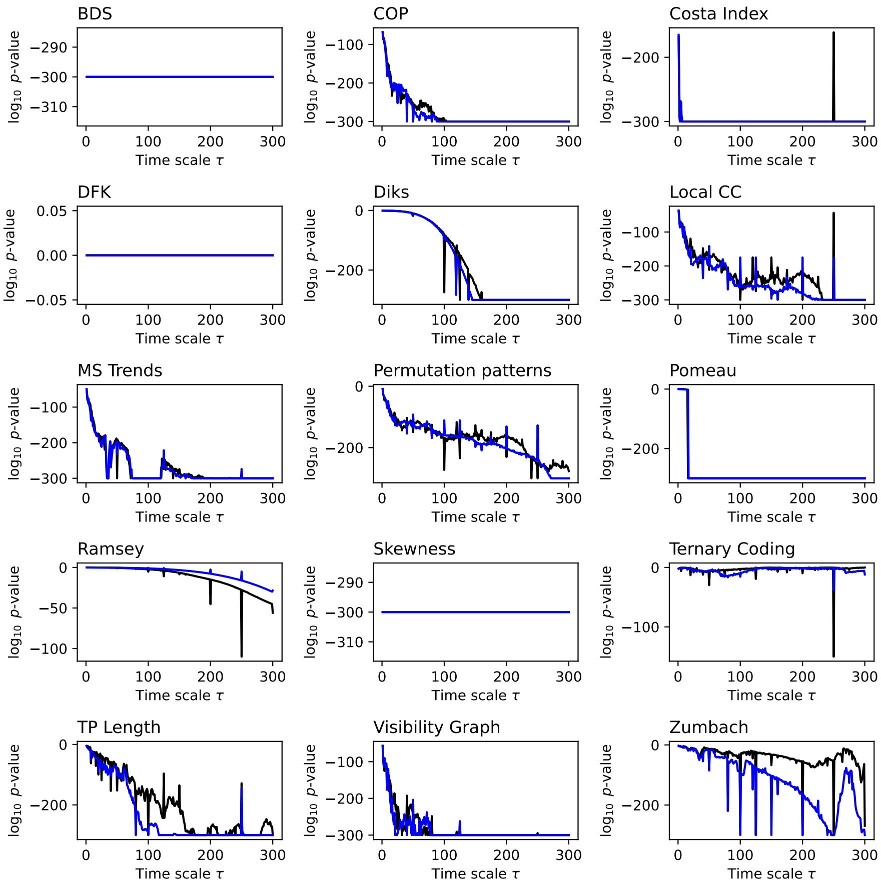

Figure S3: Evolution of the irreversibility for the asymmetric Weierstrass dynamical system (p-value). Each panel reports the evolution of the $\log_{10}$ of the p-value yielded by each test, as a function of the downsampling τ. Black lines correspond to a 1:N downsampling, blue lines to the average downsampling. p-values below 10^{-300} are set to that value. All results correspond to the median over 200 independent realisations.

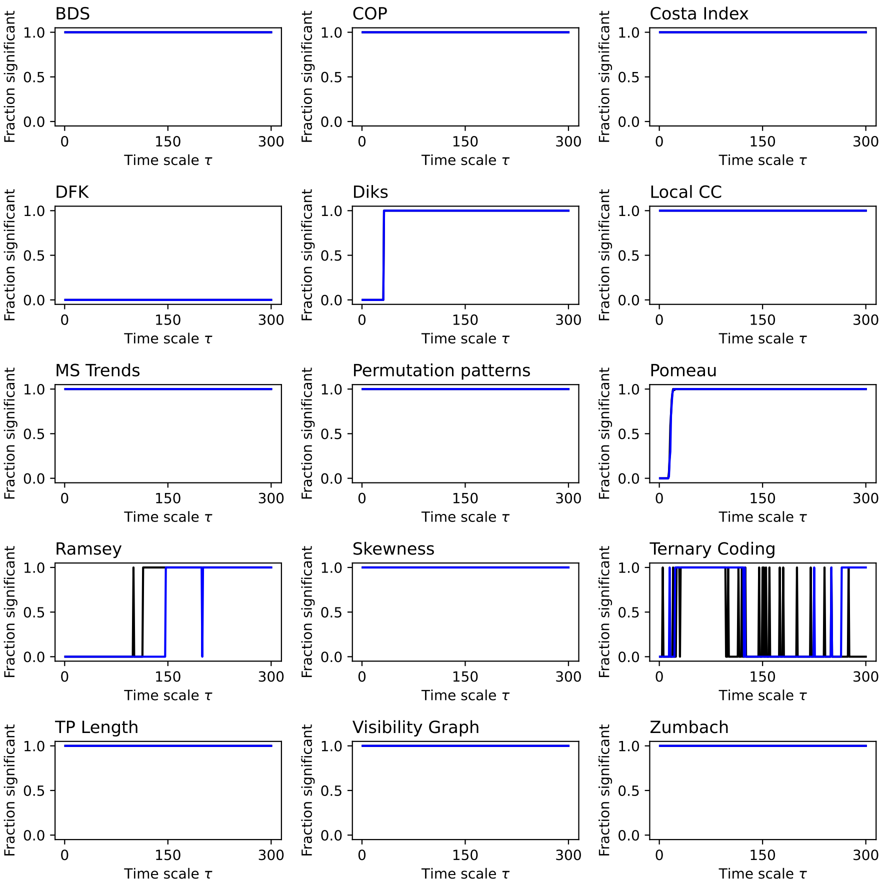

Figure S4: Evolution of the irreversibility for the asymmetric Weierstrass dynamical system (fraction). Each panel reports the evolution of the fraction of time series identified as irreversible in a statistically significant way (p-value $< 10^{-3}$) by each test, as a function of the downsampling τ. Black lines correspond to a 1:N downsampling, blue lines to the average downsampling. All results correspond to the median over 200 independent realisations.

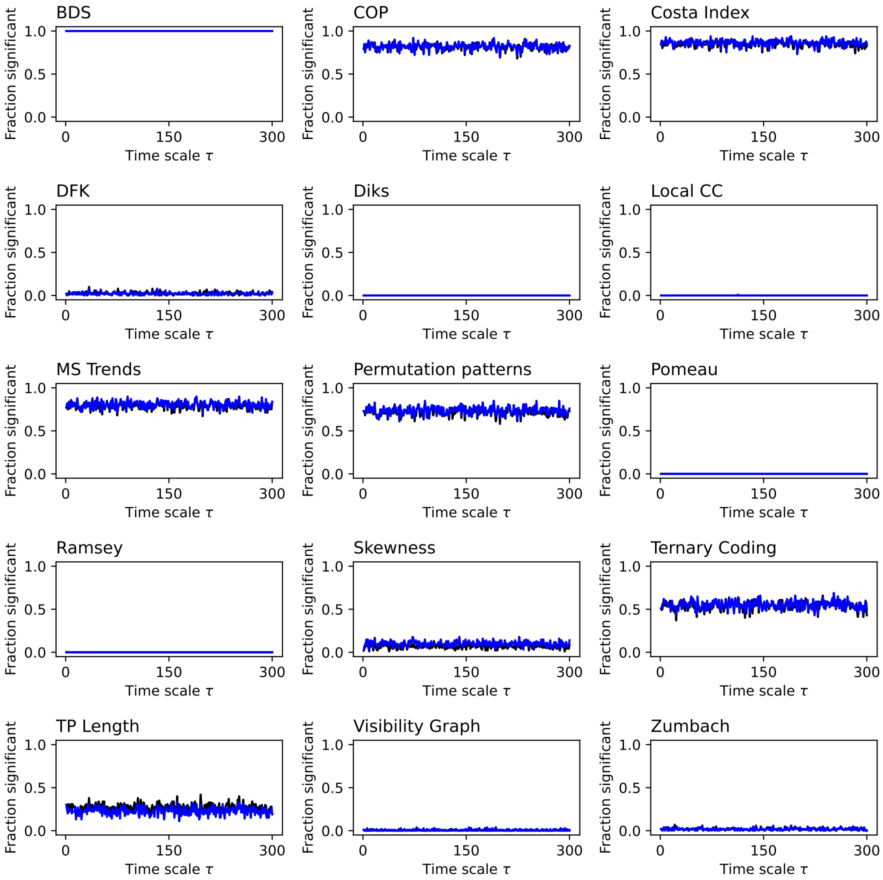

Figure S5: Evolution of the irreversibility for the fBM (fraction). Each panel reports the evolution of the fraction of time series identified as irreversible in a statistically significant way (p-value $< 10^{-3}$) by each test, as a function of the downsampling τ. Black lines correspond to a 1:N downsampling, blue lines to the average downsampling. All results correspond to the median over 200 independent realisations.

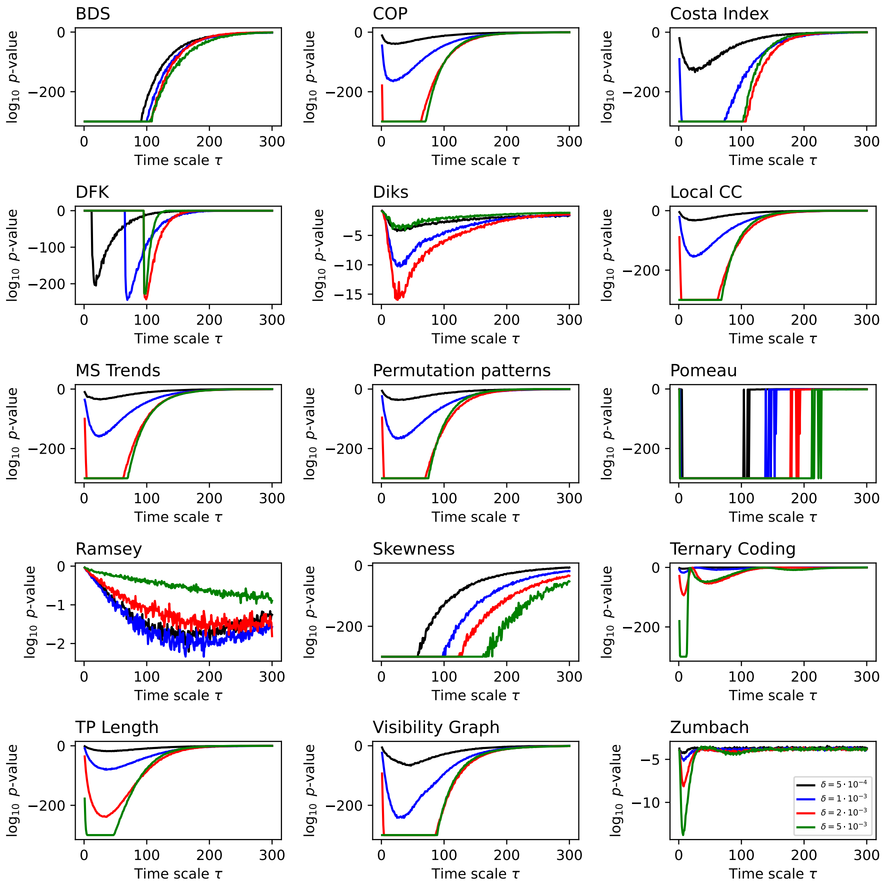

Figure S6: Evolution of the irreversibility for the srGBM dynamical system (p-value). Each panel reports the evolution of the $\log_{10}$ of the p-value yielded by each test, as a function of the downsampling τ. Black lines correspond to a 1:N downsampling, blue lines to the average downsampling. p-values below 10^{-300} are set to that value. All results correspond to the median over 200 independent realisations.

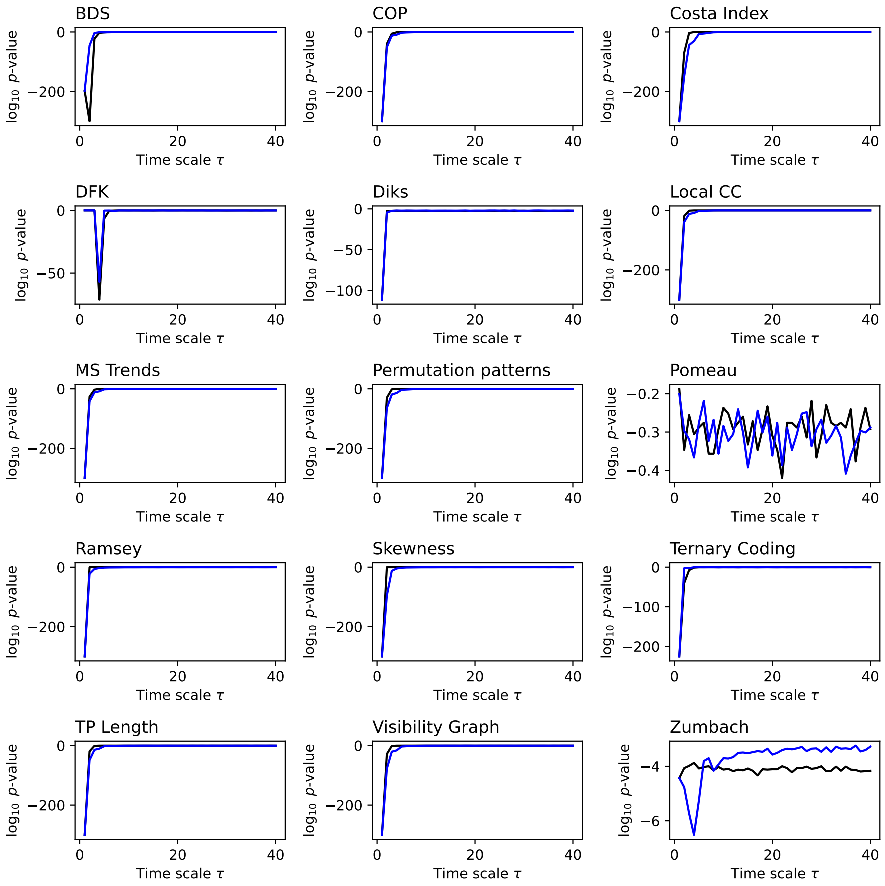

Figure S7: Evolution of the irreversibility for the Logistic map (p-value). Each panel reports the evolution of the $\log_{10}$ of the p-value yielded by each test, as a function of the downsampling τ. Black lines correspond to a 1:N downsampling, blue lines to the average downsampling. p-values below 10^{-300} are set to that value. All results correspond to the median over 200 independent realisations.

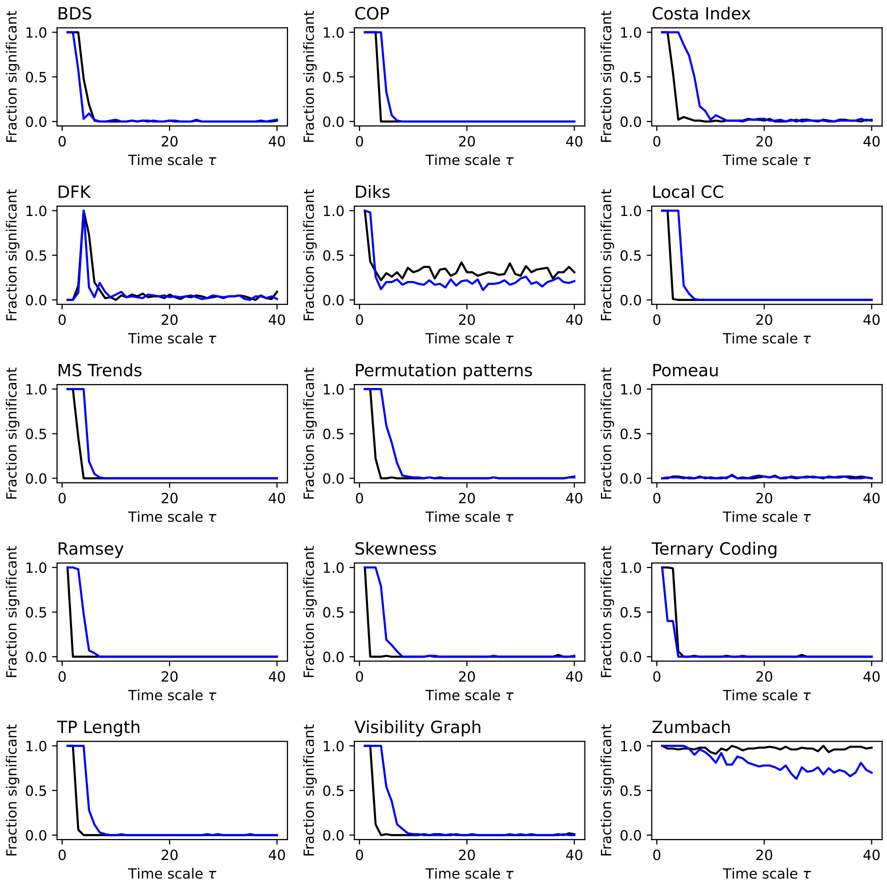

Figure S8: Evolution of the irreversibility for the Logistic map (fraction). Each panel reports the evolution of the fraction of time series identified as irreversible in a statistically significant way (p-value $< 10^{-3}$) by each test, as a function of the downsampling τ. Black lines correspond to a 1:N downsampling, blue lines to the average downsampling. All results correspond to the median over 200 independent realisations.

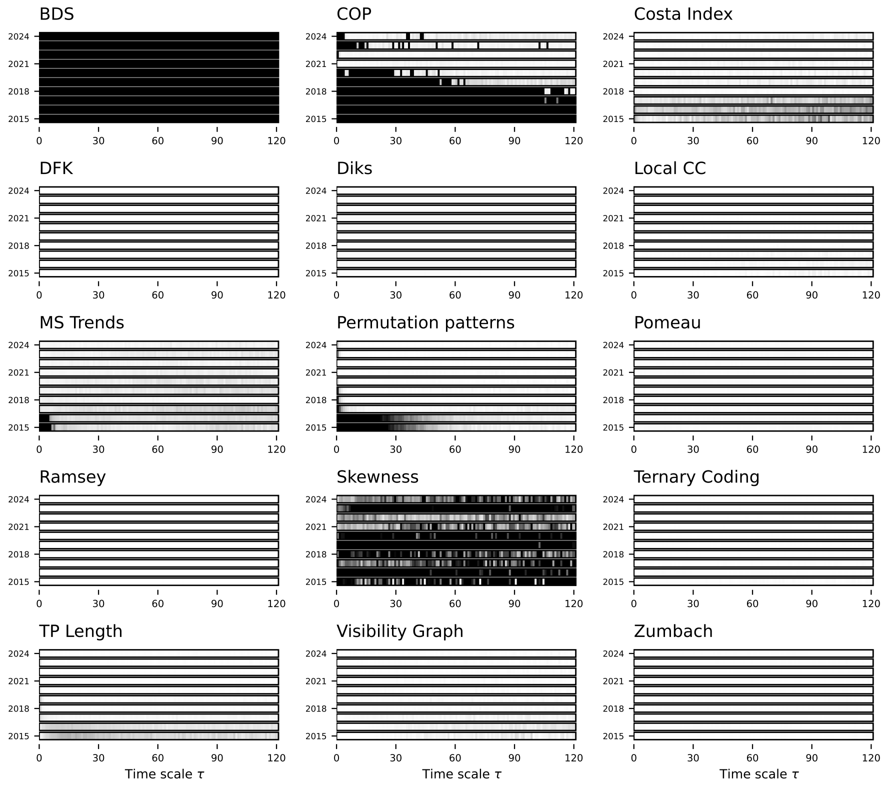

Figure S9: Evolution of the irreversibility in the Bitcoin price data. Each bar reports the evolution of the median p-value yielded by all considered tests (see name of top of panels), as a function of the downsampling τ. Additionally, each bar depicts the result for one year of data, from bottom (2015) to top (2024). Colour intensities indicate the p-value, from 1.0 (light shades, not irreversible) to 0.0 (dark shades, irreversible). All results correspond to the median over 200 independent data windows.
